# Clinical EFT as an evidence-based practice for the treatment of psychological and physiological conditions: A systematic review

**DOI:** 10.3389/fpsyg.2022.951451

**Published:** 2022-11-10

**Authors:** Dawson Church, Peta Stapleton, Anitha Vasudevan, Tom O'Keefe

**Affiliations:** ^1^National Institute for Integrative Healthcare, Petaluma, CA, United States; ^2^Department of Society and Design, Bond University, Gold Coast, QLD, Australia

**Keywords:** emotional freedom techniques (EFT), anxiety, depression, PTSD, somatic symptoms, pain, insomnia, obesity

## Abstract

**Background:**

Since the turn of the century, Emotional Freedom Techniques (EFT) has come into widespread use in medical and psychological treatment settings. It is also used as self-help by tens of millions of people each year. Clinical EFT, the manualized form of the method, has been validated as an “evidence-based” practice using criteria published by the American Psychological Association (APA) Division 12 Task Force on Empirically Validated Therapies. Its three essential ingredients are exposure, cognitive framing, and acupressure.

**Objectives:**

In 2013 we published a paper defining Clinical EFT and reviewing published research. It has been viewed or downloaded over 36,000 times, indicating widespread interest in this treatment modality. Here we update our findings based on subsequently published literature and propose directions for future research.

**Method:**

We performed a systematic review of the literature to identify randomized controlled trials (RCTs) and meta-analyses. Retrieval of 4,167 results resulted in the identification of 56 RCTs (*n* = 2,013), 41 of which were published subsequent to our earlier review, as well as eight meta-analyses.

**Results:**

RCTs have found EFT treatment to be effective for (a) psychological conditions such as anxiety, depression, phobias, and posttraumatic stress disorder (PTSD); (b) physiological issues such as pain, insomnia, and autoimmune conditions; (c) professional and sports performance; and (d) biological markers of stress. Meta-analyses evaluating the effect of EFT treatment have found it to be “moderate” to “large.” Successful independent replication studies have been carried out for anxiety, depression, PTSD, phobias, sports performance, and cortisol levels. We outline the next steps in EFT research. These include determining its impact on cancer, heart disease, diabetes, and cognitive impairment; analysis of the large-scale datasets made possible by mobile apps; and delivery through channels such as virtual practitioner sessions, artificial intelligence agents, online courses, apps, virtual reality platforms, and standardized group therapy.

**Conclusions:**

Subsequent research has confirmed the conclusions of earlier studies. These find Clinical EFT to be efficacious for a range of psychological and physiological conditions. Comparatively few treatment sessions are required, treatment is effective whether delivered in person or virtually, and symptom improvements persist over time. Treatment is associated with measurable biological effects in the dimensions of gene expression, brain synchrony, hormonal synthesis, and a wide range of biomarkers. Clinical EFT is a stable and mature method with an extensive evidence base. Its use in primary care settings as a safe, rapid, reliable, and effective treatment for both psychological and medical diagnoses continues to grow.

## Introduction

Emotional Freedom Techniques (EFT) is an “evidence-based” therapeutic method (Church, 2013a). It combines elements of cognitive and exposure therapy with acupressure. It is popularly termed “tapping” because its distinguishing feature is the stimulation of acupuncture points using fingertip percussion. EFT (Craig, 2008/2010; Church, 2018) is a simplified method of an earlier innovation termed Thought Field Therapy (TFT) developed by clinical psychologist Roger Callahan (Callahan, 1985). Though Callahan popularized tapping on acupressure points in the 1980s, he learned the method from others (Diamond, 1985; Goodheart, 1987) and tapping itself has been used in Chinese medicine, Japanese massage, qigong, and yoga for thousands of years. One of Callahan's students simplified Callahan's TFT method and described it in *The EFT Manual* (Craig and Fowlie, 1995). With instruction available free online, the modality has spread widely.

Evidence-based practices are methods that meet formally established criteria for efficacy (Beautler et al., 2005; Melnyk and Fineout-Overholt, 2005). There are several organizations that define and publish such standards. Two US government's agencies that perform this function are the Food and Drug Administration (Food and Drug Administration [FDA], 1998) and the Institute of Medicine (Institute of Medicine [IOM], 2008). Another is the UK government's National Institute for Health and Clinical Excellence (National Institute for Health and Clinical Excellence [NICE], 2009). Between 1996 and 1998 an influential set of standards in the field of psychology was published by the Task Force on Empirically Validated Treatments set up by Division 12 (Clinical Psychology) of the American Psychological Association (APA; Chambless et al., 1996, 1998; Chambless and Hollon, 1998). For convenience these are referred to as “APA standards.”

Updates to the standards have been proposed (Tolin et al., 2015). These have been implemented in the most recent APA practice guideline for Post Traumatic Stress Disorder (PTSD) (Courtois et al., 2017). However, errors in the guideline (Dominguez and Lee, 2017) and controversy surrounding the entire approach to updating the standards (Norcross and Wampold, 2019) have made their wider acceptance uncertain. Virtually all of the studies reviewed in this paper were designed while the original standards were in effect, and consensus has not been reached on updates to the standards, so we continue to refer to the original standards (Chambless et al., 1996, 1998; Chambless and Hollon, 1998) as the “APA standards.”

### The need for a definition of clinical EFT

Several million people worldwide practice EFT (Feinstein, 2021). Sources for virtual EFT such as websites, online events, and apps attract millions of people annually. Reports from Google Analytics, Semrush, and WebsiteIQ.com, tools for analyzing web traffic, found that in the last quarter of 2021, a monthly average of 336,674 people visited the top four EFT websites using computers and smartphones. Over a million subscribers had opted in to receive the newsletters published by these sites. As of 2022, over three million individuals had downloaded the self-help instructions for EFT, *The EFT Manual* (Craig and Fowlie, 1995) and *The EFT Mini-Manual* (Church, 2009/2013). An annual virtual conference called the Tapping World Summit entered its 14th year in 2022. It attracted 605,355 participants (personal communication, Nick Ortner, March 11, 2022). The most popular EFT app is called the Tapping Solution. Between its introduction in 2018 and mid-2022, over 2,107,000 users downloaded the app, while over 10 million sessions were recorded (personal communication, Nick Ortner, March 11, 2022). Available data indicates that, worldwide, tens of millions of people use EFT.

EFT is also practiced in medical and mental health settings. A survey of 149 licensed psychotherapists found that 42% were using or had considered using acupressure-based techniques similar to EFT (Gaudiano et al., 2012). In 2017, the Integrative Medicine office of the US Veterans Administration designated EFT as a “generally safe therapy” and listed it as such on the VA intranet (Church, 2017). Numerous licensed mental health professionals within the VA have trained in EFT and use it with their clients. EFT is taught or used with patients in other hospital systems such as Kaiser Permanente in the US and the National Health Service in the UK. There are over 90 EFT studies published in non-English-language journals and these demonstrate its use in non-Western countries in many conventional medical settings such as hospitals, universities, and clinics (Freedom et al., 2022).

While EFT is used in many professional settings, the number of sessions recorded on EFT websites and in the tapping app makes it apparent that the majority of its use is as self-help. While the skillful and therapeutic use of the many EFT techniques requires extensive training, its basic tapping routine is easily learned; *The EFT Mini-Manual* (Church, 2009/2013) concludes with “EFT on a Page.”

There are several professional organizations that offer training and certification in EFT. However, there is no central organization defining EFT and controlling its intellectual property, as is the case with EMDR (Eye Movement Desensitization and Reprocessing), Sensorimotor Psychotherapy, and many other modalities. Many of those who learned EFT informally created their own unique versions of the method (Feinstein, 2009). Few present the original EFT method as it is detailed in the manual (in four editions, Craig and Fowlie, 1995; Craig, 2008/2010; Church, 2013b/2018). This resulted in considerable confusion as to what EFT actually was and, in turn, to the need for a formal definition of EFT. This led in 2013 to a formal consensus paper. It defined Clinical EFT as the evidence-based manualized method that has been validated in research studies that meet the APA standards defined below (Church, 2013a).

Research studies conforming to these standards typically use a manual, *The EFT Manual* (Church, 2013b/2018; Craig, 2008/2010), and employ fidelity checks to ensure that practitioners apply EFT as described in the manual. Training of practitioners is performed using the precise methods described in the manual and validated in research.

Clinical EFT identifies 48 distinct techniques described in the manual and supplementary materials (www.ClinicalEFT.com). Clinical EFT includes techniques from Cognitive Behavioral Therapy (CBT) and Prolonged Exposure therapy (PE). These include awareness building, imaginal exposure, cognitive reframing, preframing, and systematic desensitization. To this it adds the novel ingredient of acupressure. Rather than using acupuncture needles, practitioners stimulate acupuncture points by tapping on them with their fingertips. For this reason EFT is popularly referred to as “tapping.” The addition of acupressure to established psychological techniques has been found in a meta-analysis to contribute to EFT's therapeutic results (Church et al., 2018b).

Clinical EFT was formally defined in an earlier paper by the first author (Church, 2013a). The need for a definition of Clinical EFT is demonstrated by the interest in that paper. It has been cited over 100 times and been viewed or downloaded from the journal's website over 36,000 times (personal communication, Ray Wong, November 11, 2021). In the decade since that publication, the number of studies has more than doubled, while several meta-analyses and review papers have been published. For this reason, this update of the earlier paper has been undertaken.

The current paper differs from the previous paper in several regards. Increased research has resulted in a better-defined picture of EFT's therapeutic effects. Since the previous paper was published in 2013, eight systematic reviews with meta-analyses have been performed (see Table 1). While the earlier paper summarized key studies, this update focuses on meta-analyses where available.

**Table 1:** EFT systematic reviews with meta-analyses published since 2013.

**S.R.No**.	**Condition**	**Study name**
1	Anxiety	Clond (2016). Emotional Freedom Techniques for anxiety.
2	Depression	Nelms and Castel (2016). A systematic review and meta-analysis of randomized and nonrandomized trials of clinical Emotional Freedom Techniques (EFT) for the treatment of depression.
3	PTSD	Sebastian and Nelms (2017). The effectiveness of Emotional Freedom Techniques in the treatment of posttraumatic stress disorder: A meta-analysis.
4	PTSD	Mavranezouli et al. (2019). Psychological and psychosocial treatments for children and young people with posttraumatic stress disorder: A network meta-analysis.
5	PTSD	Mavranezouli et al. (2020). Psychological treatments for post-traumatic stress disorder in adults: A network meta-analysis.
6	Somatic symptoms	Stapleton et al. (2021). Emotional Freedom Techniques (EFT) for somatic symptoms: A systematic review and meta-analysis.
7	Pain, anxiety, depression, burnout, stress, phobia	Church et al. (2018b). Is tapping on acupuncture points an active ingredient in Emotional Freedom Techniques? A systematic review and meta-analysis of comparative studies.
8	Pain, anxiety, depression, PTSD, food cravings, phobia	Gilomen and Lee (2015). The efficacy of acupoint stimulation in the treatment of psychological distress: A meta-analysis.

Sr. No., systematic review number.

For conditions for which meta-analyses are not available, such as phobias and weight loss, we draw on the evidence provided by individual studies, preferably RCTs. The earlier paper called for more research into EFT's physiological mechanisms of action and such research is now available. While many of the studies in the earlier paper were performed by practitioners, current research often involves universities, granting agencies, governments, and institutes. While a review paper like this is limited to the studies published to date, additional studies continue to be published. A current list is maintained at Research.EFTuniverse.com.

### APA standards

The original APA standards were defined in a series of papers (Chambless et al., 1996, 1998; Chambless and Hollon, 1998). Therapies demonstrating efficacy according to certain criteria, such as two high-quality studies performed by independent investigators finding the method statistically superior to a placebo or another method, are said to be “efficacious.” Methods that meet lesser standards are classified as “probably efficacious.”

The APA standards may be summarized as comprising seven essential criteria (Energy Psychology, 2017) and studies are deemed “empirically validated” if they meet all seven. Chambless and Hollon (1998) also list additional criteria designated as “highly desirable” or “desirable”. The seven essential criteria are:

1. **Randomized controlled trials** (RCTs)—subjects were randomly assigned to the treatment of interest condition or to one or more comparison conditions.
2. **Adequate sample size** to detect statistically significant (*p* < 0.05 or better) differences between the treatment of interest and the comparison condition(s) was used.
3. **The population for which the treatment was designed and tested must be clearly defined** through the use of diagnosis by qualified clinicians, through cutoff scores on questionnaires that are reliable and valid, through interviews identifying the focus of the study's interest, or through some combination of these.
4. Assessment tools must have demonstrated **reliability and validity** in previous research.
5. Any interview assessments were made by interviewers who were **blind to group assignment**.
6. **Treatment manuals** that make clear the nature of the treatment being tested were used. If the treatment was relatively simple, it could be described in the procedure section of the journal article presenting the experiment, in lieu of a treatment manual.
7. The paper reporting the study **provided enough data** that the study's conclusions can be reviewed for appropriateness, including sample sizes, use of instruments that detect changes targeted by the study's design, and **magnitude of statistical significance**.

Studies of efficacious or probably efficacious therapies are required to demonstrate “statistically significant” results, meaning there is < 1 possibility in 20 (i.e., 0.05%) that the results are due to chance (Criterion #2). Statistical significance is defined as *p* < 0.05. The term “highly significant” is often used to refer to studies with < 1 possibility in 1,000 that the results are due to chance, or *p* < 0.001.

Revisions to the APA standards were proposed in 2015 (Tolin et al., 2015) and used in a treatment guideline for PTSD (Courtois et al., 2017). The standards by which treatments were assessed were made extremely rigorous, such as prioritizing the number and quality of RCTs and meta-analyses. However, these revisions drew criticism for the obvious reason that older therapies have more studies, while newer therapies (which might be innovative and effective) have fewer. Norcross and Wampold (2019, p. 393) observed: “The difference in recommendations resides in the number of RCTs conducted on each treatment. If numbers are good, more numbers must prove better! We understand the decision to elevate those trauma psychotherapies that possess more studies—‘strength of evidence'—to the category of strongly recommended. However, at the risk of stating the obvious, more studies do not mean more effectiveness…. Practitioners seek what is effective for their patients, not what is most studied.” Division 12 maintains an online list of treatments (American Psychological Association [APA], 2021) based on both the original APA standards and the revised standards (Tolin et al., 2015).

For more than a quarter-century, the original APA criteria (Chambless et al., 1996, 1998; Chambless and Hollon, 1998) have provided a stable, well-defined, published set of common standards by which the efficacy of a therapeutic technique may be judged. When that technique is then translated into training, certification, and clinical practice, these criteria provide reasonable assurance that the method as practiced in the field is the method that has been validated in research. A 1-year certification program that trains practitioners in the 48 Clinical EFT techniques has been offered since 2010 (UltimateEFTcertification.com).

Most of the meta-analyses summarized in Table 1 above used the APA criteria as a quality control. The RCTs performed subsequent to the meta-analyses and reported in this paper are not evaluated against the APA criteria because no statistical analysis of their results has been performed and because a primary goal of this review is to make the entire evidence base easily available and comprehensible to clinicians.

### EFT as an empirically validated treatment

Having defined Clinical EFT and identified the set of standards upon which measurement of efficacy is based, we can now examine the evidence base for the method. The first group of EFT studies performed were outcome studies. Outcome studies use experimental designs that highlight participant outcomes, asking the question “Are participants better off after treatment?” We examine studies demonstrating the efficacy of Clinical EFT for:

- Psychological conditions such as PTSD, phobias, depression, and anxiety;
- Physiological problems such as pain and autoimmune conditions; and
- Performance in sports, business, and academic pursuits.

We also summarize the key research on the physiological mechanisms of action of Clinical EFT, showing how EFT works in the body to effect change. These studies, rather than measuring whether treatment benefits patients, ask the questions characteristic of basic science, such as “How does this treatment work?” and “What is changing in the body as a result of this treatment?”

The final group of studies reviewed investigates EFT's application to performance issues such as business and sports performance. We also examine the evidence for whether EFT's somatic component, tapping with the fingertips on acupressure points, is an inert placebo or an active ingredient in the results obtained.

**Figure 1:**
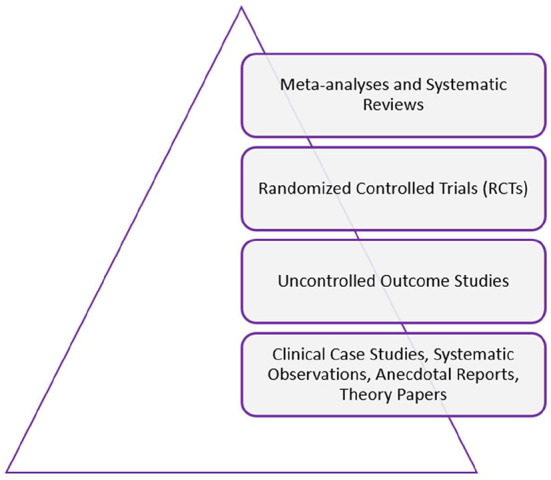
Hierarchy of evidence.

As shown in Figure 1, we use the “hierarchy of evidence” model, which places meta-analyses and systematic reviews at the top of a pyramid of evidence (Feinstein, 2021). In the tier below these come RCTs and then uncontrolled outcome studies. Below these are clinical case studies, systematic observations, anecdotal reports, and theory papers.

To provide a measure of clinical significance and show the magnitude of the effect of EFT treatment, we use the Cohen's *d* or Hedge's *g* statistic. On this scale a score of 0.2 indicates an observable treatment effect, 0.5 a moderate effect, and 0.8 a large effect.

In cases where important studies were published after the date of a meta-analysis, we catalog them to bring the evidence base current. We also note whether meta-analyses were performed by proponents of EFT or by independent statisticians with no stated conflict of interest. If a paper was written by proponents, but the statistician was independent, that distinction is clarified.

Finally, we derive the meaning of this whole body of work and extend it to show the next steps in EFT research and clinical practice. These include extending its accessibility through apps, its applicability to personalized medicine, the use of new technologies such as virtual reality (VR) and artificial intelligence (AI), the analysis of the huge datasets made available by apps, the accessibility advantages of video treatment, the utility of online and virtual courses, and Clinical EFT's widespread adoption in primary care.

### Replications

Studies that explicitly set out to replicate earlier studies are highlighted. There has been a great deal of discussion in the research community about the “replication crisis” in science (Kaiser, 2017). Shortly after the turn of this century, the multinational biotech company Amgen set out to replicate 53 “landmark” studies on which it planned to base its next generation of cancer drug development. The company was able to replicate only six. An analysis in the journal *Nature* characterized this “a shocking result” (Begley and Ellis, 2012). Another pharma company, Bayer, had similar results. An attempt to replicate five cancer biology trials was successful for only two (eLife, 2017; Kaiser, 2017).

The paucity of replicable results is similar in the social sciences. An international group of 270 investigators set out to replicate 100 studies published in 2008 in three top psychology journals. They found that they were able to replicate fewer than half of them (Open Science Collaboration, 2015).

The journal *Nature* also conducted a survey of 1,576 researchers to identify their experiences with replication. It found that over 70% of them had failed when attempting to reproduce another scientist's research findings. Over half could not even replicate their own research (Baker, 2016).

For this reason, we identify studies that were explicitly designed as replications or extensions of earlier research and note similarities and differences between the findings of the replications and the original studies.

## Methods

### Systematic review procedure

For this systematic review, the fourth author conducted searches in three online databases: PsychINFO, Medline/Pubmed, and EBSCO Essentials. Search terms limited the results to peer-reviewed English-language professional journals. The search terms “EFT,” “Emotional Freedom Technique” (singular), and “Emotional Freedom Techniques” (plural) were used. Allied and hybrid methods such as Thought Field Therapy (TFT) and Spiritual EFT (SEFT) were excluded.

We ranked studies using the “hierarchy of evidence” approach (Feinstein, 2021). Where a meta-analysis that included RCTs was available, no RCTs included in that meta-analysis were included in our results, only RCTs published after that date.

To satisfy the inclusion criteria based on the aims of the review, two initial searches were conducted of the PsychINFO and PubMed databases on April 25, 2022, followed by a third database (EBSCO) on April 28, 2022. The PsychINFO and PubMed searches used the following terms: ((EFT) OR (Emotional Freedom Technique) OR (Emotional Freedom Techniques)) NOT (Thought Field Therapy) NOT (Spiritual EFT). Results were filtered by “Meta-Analysis, Randomized Controlled Trial, Systematic Review, Validation Study, English” in PubMed, resulting in 187 articles and “English Language, Peer Reviewed Journal” in PsychINFO, resulting in 3,830 articles. The latter EBSCO Essentials search, using the term “Emotional Freedom Technique” filtered by “Academic (Peer-Reviewed) Journals, English” and conducted as a title search, resulted in 150 articles. A checklist comparing the reporting of this systematic review with the standards of the Preferred Reporting Items for Systematic Review and Meta-Analysis (PRISMA) Statement is available as a Supplementary File.

The final results returned a total of 4,167 studies for consideration. After removing duplicates and ineligible studies, abstract and in-text screening was performed. The reference lists of included studies were manually reviewed by the fourth author for additional studies. This resulted in a final total of 139 studies, 56 of which were RCTs. Of these, 41 had been published subsequent to the available meta-analyses. Where uncertainty about including a study existed, the other three authors were consulted until consensus had been reached.

Many studies of EFT have been conducted in non-English-speaking countries. A systematic review of these, conducted in October 2021, identified 91 studies published in non-English-language journals (Freedom et al., 2022). While the inclusion criteria for the current investigation excluded this body of literature, it is noteworthy that EFT is being investigated in many different parts of the world other than English-speaking countries.

## Results

### Psychological health outcome studies

Clinical EFT has met APA standards as an “efficacious” treatment for a number of conditions, including anxiety, depression, phobias, and PTSD (Church et al., 2018b). Meta-analyses of EFT for anxiety, depression, and PTSD are available. The methodology of some of these encompasses only RCTs. Others use RCTs for the primary analysis but also examine the data from uncontrolled outcome studies in a secondary analysis. Since RCTs are just below meta-analyses in the hierarchy, the sections on mental health outcomes focus on these. Occasionally, a study of particular interest that is not an RCT is described if it is particularly relevant to clinicians. Each section begins with a description of the meta-analysis if one is available for that condition. Where no meta-analyses or RCTs for a condition are available, we describe illustrative studies further down the hierarchy.

#### Anxiety

An independent meta-analysis of EFT for anxiety was published in 2016 (Clond, 2016). Its literature review included all anxiety RCTs published up through the end of 2015 and it used the APA standards as a quality control screen. It identified 14 studies (*N* = 658). It found a “large” treatment effect size, with a Cohen's *d* = 1.23 (*p* < 0.001). Control therapies included Progressive Muscular Relaxation (PMR), Diaphragmatic Breathing (DB), and Cognitive Behavior Therapy (CBT). The effect size for the combined controls was 0.41 (*p* = 0.001), indicating that EFT produced superior treatment effects.

Among the populations studied in the 14 trials were veterans with PTSD, students suffering from presentation anxiety and test anxiety, gifted children, hospital patients, subjects with specific phobias, fibromyalgia patients, and weight loss program participants. Treatment time frames ranged from one to six sessions. Follow-ups demonstrated that participant gains were durable. Clond (2016) noted that a limitation of the meta-analysis was the small number of studies comparing EFT to a known effective treatment such as CBT. Table 2 shows the RCTs included in the meta-analysis.

**Table 2:** Anxiety RCTs included in Clond (2016) meta-analysis.

**S.R.No**.	**References**	**Population**	**Assessment tool**	**EFT (*n*) and sessions**	**Control/s (*n*) and sessions**	**dEFT – dctrl (95% CI)**	***p***
1	Baker and Siegel (2010)	31 people with specific phobia	FQ, DSM-IV	(*n* = 11) One 45-min session	1. NT (*n* = 10) 2. Interview (*n* = 10) 1 Interview session	0.83 (−0.26–1.92) 0.91 (−0.18–2.00)	*p* = 0.136 *p* = 0.102
2	Brattberg (2008)	86 women with fibromyalgia (>50% with anxiety)	HADS	(*n* = 26) 1/day for 8 weeks	WL (*n* = 36)	0.49 (−0.06–1.04)	*p* = 0.083
3	Church et al. (2013)	59 veterans with PTSD	SA-45	(*n* = 30) 6 sessions	TAU (*n* = 29)	1.52 (0.81–2.23)	*p < * 0.001^*^
4	Church et al. (2016)	21 subclinical PTSD veterans	SA-45	(*n* = 12) 6 sessions	TAU (*n* = 9)	1.18 (0.04–2.32)	*p* = 0.043^*^
5	Church et al. (2012c)	83 nonclinical subjects (Almost 50% with anxiety)	SA-45	(*n* = 28) One 1-h session	1. NT (*n* = 27) 2. Interview (*n* = 28) One 1-h supportive Interview session	1.34 (0.66–2.02) 0.71 (0.00–1.42)	*p < * 0.001^*^p = 0.049^*^
6	Fox (2013)	20 undergraduate students	AEQ	(*n* = 10) One 40-min session	Modified EFT (*n* = 10) 1 session	0.47 (−0.55–1.49)	*p* = 0.366
7	Gaesser and Karan (2017)	63 gifted children	RCMAS-2, IQ score	(*n* = 20) 3 sessions	1. WL (*n* = 21) 2. CBT (*n* = 21) 3 sessions	(0.18–2.02) 0.23 (−0.79–1.25)	*p* = 0.019^*^p = 0.658
8	Geronilla et al. (2016)	58 veterans with PTSD	SA-45	(*n* = 32) 6 sessions	TAU (*n* = 26)	2.3 (1.38–3.22)	*p* < 0.001^*^
9	Jain and Rubino (2012)	40 undergraduate students	WTAS, SA-45	(*n* = 11) 1 session	1. WL (*n* = 23) 2. DB (*n* = 6) 1 session	0.45 (−0.36–1.26) −0.73 (−2.42–0.96)	*p* = 0.275 *p* = 0.396
10	Karatzias et al. (2011)	46 NHS psychotherapy referrals with PTSD	HADS, DSM-IV	(*n* = 23) Four 1-h sessions/individual	EMDR (*n* = 23) Four 1-h sessions/individual	−0.28 (−1.16–0.60)	*p* = 0.531
11	Salas et al. (2011)	22 students meeting criteria for phobic response to specific stimulus	BAI, SUD	(*n* = 11) One session with five 2-min round of tapping	DB (*n* = 11) One session with five 2-min round of DB	0.37 (−0.63–1.37)	*p* = 0.468
12	Sezgin and Özcan (2009)	32 students with test anxiety	TAI	(*n* = 16) 1 session	PMR (*n* = 16) 1 session	1.81 (0.10–3.52)	*p* = 0.038^*^
13	Stapleton et al. (2013)	96 overweight patients (>50% with anxiety)	SA-45	(*n* = 48) One session/week for 4 weeks	WL (*n* = 48) NT for 4 weeks and then had EFT.	0.27 (−0.12–0.66)	*p* = 0.177
14	Wells et al. (2003)	35 participants with specific phobias	FQ, DSM-IV	(*n* = 18) 1 session	DB (*n* = 17) 1 session	1.64 (0.48–2.8)	*p* = 0.006^*^

* Significant results. AEQ, Achievement Emotions Questionnaire; BAI, Beck Anxiety Inventory; CBT, Cognitive Behavior Therapy; DB, Diaphragmatic Breathing; DSM-4, Diagnostic and Statistical Manual of Mental Disorders, 4th edition; EMDR, Eye Movement Desensitization and Reprocessing; FQ, Fear Questionnaire; HADS, Hospital and Anxiety Depression Scale; NHS, National Health Service; NT, no treatment; PCL-M, PTSD Checklist–Military; PMR, Progressive Muscular Relaxation; RCMAS-2, Revised Children's Manifest Anxiety Scale−2; SA-45, Symptom Assessment−45; SUD, Subjective Units of Distress; TAI, Test Anxiety Inventory; TAU, treatment as usual; WL, wait-list; WTAS, Westside Test Anxiety Scale. Sr. No., systematic review number.

Subsequent to the meta-analysis, several noteworthy new studies of anxiety have been published. An RCT of PTSD in female survivors of gender violence in the Congo assessed anxiety and depression as secondary measures, and also compared EFT to CBT (Nemiro and Papworth, 2015). Participants received two 2–1/2 h group treatment sessions per week for four consecutive weeks. Assessments occurred before and after treatment, and 6 months later. Follow-up showed that participants maintained their gains over time whether treated with EFT or CBT. The investigators used the Hopkins Symptom Checklist−25 (Derogatis et al., 1974), which includes 25 items, 15 for depression and 10 for anxiety. However, they did not report anxiety and depression separately, instead interpreting the overall score as a measure of general mental health.

After six sessions in a veterans' RCT (Church, 2014), there was a significant reduction in anxiety (*p* < 0.0001) and these gains were maintained at follow-up, after 6 months (*p* < 0.0001).

An RCT with 76 nursing students compared EFT to Breathing Therapy (BT). Both therapies were found to be effective, but the treatment effect size for EFT (*d* = 3.18) was significantly greater than that for BT (*d* = 1.46; Dincer et al., 2020). BT was also the control for an RCT of 120 pregnant women (Vural and Aslan, 2019). It found that those in the EFT group demonstrated significantly greater ability to release the pain and fear associated with labor.

Somatic anxiety and psychological anxiety were evaluated in an RCT of 50 women awaiting surgery. They received two 10-min EFT sessions, one on the day prior to surgery and the second session on the day of surgery. Anxiety scores in the EFT group dropped from 27.28 (±2.47) to 7.60 (±2.00) and were highly statistically significant (*p* < 0.0001; Thomas et al., 2017). An RCT examined the changes in anxiety levels in 60 nursing students (Inangil et al., 2020). EFT and Music Therapy (MT) were compared to a no-treatment control. It found that both EFT and MT decreased anxiety levels and that there was no statistically significant difference between their effects.

An RCT performed in India (Jasubhai and Mukundan, 2018) screened patients for stress, anxiety, depression, short-term memory, and psychophysiological coherence. Those who presented with clinical levels were randomized into two groups and given eight weekly sessions of either EFT or CBT. Follow-ups were performed after 6 weeks, 5 weeks, 1 month, and 6 months. EFT treatment produced marked improvement in depression after three sessions. After 8 weeks of intervention, the CBT group reported significant improvement (*p* < 0.05) in depression and short-term memory, while the EFT group showed significant improvement (*p* < 0.05) in depression at the 1- and 6-month follow-up points. Examination of individual cases showed clinically significant improvements in stress, anxiety, depression symptoms, short-term memory, and psychophysiological coherence across both interventions. The results are consistent with a previous study (Chatwin et al., 2016) of which the Indian study was designed as a replication.

EFT was compared to Systematic Desensitization (SD) in 16 students with high levels of public speaking anxiety (Madoni et al., 2018). The RCT concluded that both treatments were effective. However, when measured by the average result and the effect of time, the EFT decreased anxiety more than SD. The longer the duration of EFT treatment, the more anxiety decreased (pretest, *p* < 0.01; posttest, *p* < 0.001; follow-up, *p* < 0.01).

Another assessed the anxiety levels of 83 patients in treatment for obesity. It compared two 8-week programs, one EFT and the second using CBT. The CBT group did not demonstrate any significant changes in anxiety scores over time. In the EFT group, anxiety decreased significantly, and participant gains were maintained at 6- and 12-month follow-up (Stapleton et al., 2017).

In an RCT with a population of 63 high-ability students aged 10–18, EFT was also compared to CBT. They received three individual sessions with one of the two modalities. A waitlist served as control. The effect size for EFT was large, with *d* = 0.74. CBT participants also showed reductions in anxiety but did not differ significantly from the EFT or control groups (Gaesser and Karan, 2017).

In an RCT for test anxiety in Turkish nursing students preparing for their clinical exam, both music therapy (MT) and EFT led to a decrease in anxiety scores (*p* < 0.05; Inangil et al., 2020). An RCT by Kwak et al. (2020) studied anxiety in Hwabyung patients using EFT and Progressive Muscle Relaxation (PMR). Hwabyung is a psychosomatic diagnosis used in Korea to identify the suppression of anger over an extended period. It is associated with increased incidence of cancer, hypertension, and other major diseases. Patients received 4 weeks of group sessions with either EFT or PMR. Follow-ups were performed at 4 and 24 weeks. Both the EFT (*n* = 15) and PMR group (*n* = 16) demonstrated decreased Hwabyung symptoms (−13.95 and −11.46%, respectively) and state anxiety (−12.57 and −12.64%, respectively). Similarly, in an RCT measuring aggression in single mothers (Abdi and Abolmaali, 2015), symptoms were reduced after six EFT sessions (*p* < 0.01).

In a German RCT (König et al., 2019) where the treatment given to anxiety patients was either EFT or PMR, anxiety levels on the NAS scale dropped significantly in the total sample from pre to posttest (*p* = 0.001). This reduction could be shown in both intervention groups when calculating *t*-tests within both groups (EFT, *p* = 0.033; PMR, *p* = 0.013).

An Australian RCT with 168 chronic pain participants (Stapleton, 2022), which provided EFT as both online and face-to-face treatment, showed significant reduction in anxiety from pre to 6 months (*p* < 0.001). An RCT by Dincer et al. (2020) investigated the efficacy of a brief online EFT session in the prevention of stress, anxiety, and burnout among nurses involved in the treatment of COVID patients. It was conducted in the COVID department of a university hospital in Turkey and was designed using the Consolidated Standards of Reporting Trials (CONSORT) guidelines. Reductions in anxiety reached high levels of statistical significance for the intervention group (*p* < 0.001). The control group showed no statistically significant changes (*p* > 0.05).

Table 3 shows RCTs published between the date of the Clond (2016) meta-analysis and April 2022.

**Table 3:** Anxiety RCTs published since the Clond (2016) meta-analysis.

**S. No**.	**References**	**Population**	**Instrument**	**EFT (*n*) and sessions**	**Control(s) (*n*) and sessions**	***p* (EFT)**
1	Church (2014)	59 veterans with PTSD	SA-45	(*n* = 30) 6 sessions	WL (*n* = 29) NT	*p* ≤ 0.0001 (at both pretest and 6 month follow-up)
2	Nemiro and Papworth (2015)	50 female survivors of sexual gender violence in Congo	HTQ, HSC-25	(*n* = 25) Two 2.5 h group treatment/week for 4 weeks	CBT (*n* = 25) 2 group treatments/week for 4 weeks	No significant difference between EFT and CBT
3	Chatwin et al. (2016)	10 community members with MDD	DASS-21	(*n* = 6) 1 session/ week for 8 weeks	CBT (*n* = 4) 1 session/week for 8 weeks Community sample (*n* = 57)	*p* = 0.871 (same as CBT) *p* = 0.005 (compared to Community)
4	Thomas et al. (2017)	50 women awaiting surgery	MHARS	(*n* = 25) Two 10-min sessions +TAU	Control group, TAU (*n* = 25)	Anxiety scores (*p < * 0.0001), Psychological and somatic anxiety scores (*p < * 0.002)
5	Stapleton et al. (2017)	83 overweight or obese adults	PHQ	(*n* = 51) 1 session/week for 8 weeks	CBT (*n* = 34) 1 session/week for 8 weeks	6 and 12 month follow-up *p* = 0.001 and *p < * 0.001. No significant changes in CBT group at 6 and 12 months
6	Gaesser and Karan (2017)	63 high-ability students aged 10–18 yrs	RCMAS-2	(*n* = 20) 3 sessions	CBT (*n* = 21) 3 sessions WL (*n* = 21)	Compared to WL: *p* = 0.005 Compared to CBT: *p* = 0.18
7	Jasubhai and Mukundan (2018)	10 community members with MDD	DASS-21	(*n* = 10) 8 sessions	1. CBT (*n* = 10) 8 sessions 2. Control group (*n* = 57)	Pre and post EFT DASS-21 anxiety: *p* = 0.02
8	Inangil et al. (2020)	120 nursing students with situational anxiety	SAS and TVSF	(*n* = 30) One 20-min session	Breathing exercise (*n* = 30) One 20-min session Music therapy (*n* = 30) One 20-min session Control group (*n* = 30) 20-min free time	*p < * 0.05
9	Madoni et al. (2018)	16 students with high levels of public speaking anxiety	PRPSA	(*n* = 8) 2 group sessions	Systematic Desensitization (*n* = 8) 2 group sessions	Pretest (*p < * 0.01) Posttest (*p < * 0.001) Follow-up (*p < * 0.01)
10	Vural and Aslan (2019)	120 pregnant women with pain and fear associated with labor	SUD, WDEQ (version B)	(*n* = 35) 15-min demo followed by 9 EFT sessions with each pregnant woman	1. Breathing Awareness (BA) (*n* = 35) 10-min demo followed by women doing BA on their own as long as they wanted 2. Control group (*n* = 50)	Group score difference (*p < * 0.001)
11	Stapleton et al. (2019c)	282 people with food cravings and obesity	PHQ	(*n* = 145, at end of 12 months) 1 online group session/week for 8 weeks	Wait-list control group (*n* = 137) NT	Anxiety score after 8 weeks *p* = 0.012 12 mo postintervention *p < * 0.001
12	Dincer et al. (2020)	78 nursing students with public speaking anxiety	SUD, STAI, Speech Anxiety Scale	(*n* = 25) 1 session with 3 rounds of tapping, 3 min each	Breathing therapy (*n* = 26) *One* instructional session with practice Control group (*n* = 25) NT	*p < * 0.001
13	Inangil et al. (2020)	90 nursing students with test anxiety	SAS and TVSF	(*n* = 30) 1 session with 3 rounds of tapping, 3 min each	1. Music therapy (*n* = 30) 15 min 2. Control group (*n* = 30) NT	*p < * 0.05
14	Baghini et al. (2020)	60 male PTSD patients with anxiety	SGLVJAI	(*n* = 15) Six 60-min sessions	EMDR (*n* = 15) Six 45-min sessions CBT (*n* = 15) Six 60-min sessions Control group (*n* = 15) NT	*p < * 0.02
15	Kwak et al. (2020)	31 Hwabyung patients	TSH, VAS-HS, BDI, STAI and STAXI	(*n* = 15) 4 group sessions	PMR (*n* = 16) 4 group sessions	*p < * 0.05
16	Dincer and Inangil (2020)	72 nurses caring for COVID-19 patients	STAI-SAS, SUD, BI	(*n* = 35) 1 guided online group session	NT control group (*n* = 37)	*p < * 0.001
17	Abdi and Abolmaali (2015)	30 single mothers with high aggression	AAT	(*n* = 15) 12 EFT sessions	NT control group (*n* = 15)	*p < * 0.01
18	König et al. (2019)	22 anxiety patients	NAS score	(*n* = 9) One 60-min session	PMR (*n* = 13) One 60-min session	Pre to posttest EFT *p* = 0.033 PMR *p* = 0.013
19	Stapleton (2022)	168 chronic pain patients	PHQ	(*n* = 91) 6-week live facilitator led (*n* = 90) Self-paced online program	WL for live group (*n* = 45) WL for self paced (*n* = 50) WL group was later given EFT	Pre to 6-month *p* < 0.001

AAT, Abolmaali Aggression Test; BDI-2, Beck Depression Inventory, 2nd edition; BI, Burnout Inventory; CBT, Cognitive Behavior Therapy; DASS-21, Depression, Anxiety, and Stress Scale−21; HSC-25, Hopkins Symptom Checklist−25; HTQ, Harvard Trauma Questionnaire; ICD-10, International Classification of Diseases 10th revision; MDD, Major Depressive Disorder; MHARS, Modified Hamilton Anxiety Rating Scale; NAS, Numeric Analog Scale; NT, no treatment; PHQ, Patient Health Questionnaire; PMR, Progressive Muscular Relaxation; PRPSA, Personal Report of Public Speaking Anxiety; RCMAS-2, Revised Children's Manifest Anxiety Scale−2; SAS, Situational Anxiety Scale; SGLVJAI, Spielberger, Gorsuch, Lushene, Vagg, & Jacobs's Anxiety Inventory; STAI, State-Trait Anxiety Inventory; STAI-SAS, State-Trait Anxiety Inventory–State Anxiety Scale; STAXI, State-Trait Anger Expression Inventory; SUD, Subjective Units of Distress; TAU, treatment as usual; TSH, The Hwabyung Scale; TVSF, The Vital Signs Form; VAS-HS, Visual Analog Scale of Hwabyung Symptoms; WDEQ, Wijma Delivery Expectancy/Experience Questionnaire.

Three studies that are not RCTs deserve mention. The development of smartphone apps has presented new opportunities for gathering data. The dominant EFT app is called the Tapping Solution. Between October 2018 and October 2019, data were gathered from 270,461 app users (Church et al., 2020a). It was found that across 12 tapping meditations targeting anxiety and stress, users reported a 44% reduction in symptoms (*p* < 0.001). This study illustrates the power of smartphone data collection, which allows the participation of hundreds of thousands of participants, as well as the efficacy of EFT when practiced in this format.

The second uncontrolled study took the form of a “service evaluation” in a clinic in Britain's National Health Service (Boath et al., 2014a). It examined patient acceptance of EFT as well as EFT's success in reducing symptoms. It identified a significant improvement in anxiety, with a mean treatment time frame of eight sessions. It also found a significant improvement in overall psychological health and physical functioning. This is one of a number of studies applying EFT in primary care settings.

The final study is also not an RCT but worth mentioning because it is the study most often cited by EFT's critics. Waite and Holder (2003) compared EFT to two sham tapping interventions and a non-tapping control group. One control group tapped on points not specified in the EFT protocol. The second tapped on a doll. Statistically significant improvements were found in all three tapping groups but not in the control group. The authors concluded that because those who tapped on other points or the doll improved, “certain components of EFT were effective, but not dependent on meridian points as EFT supporters contend.” They interpreted their findings to mean: “It is possible that systematic desensitization and distraction are mediators of EFT's apparent effectiveness” (p. 24).

The study suffered from a number of design limitations, however. It was not randomized (APA Criterion #1). It failed to use valid and reliable assessments (APA Criterion #4), failed to apply EFT with fidelity to the manual (APA criterion #6), and failed to recognize that the “sham” points chosen were in fact actual acupressure points (APA criterion #6). Reappraisals of the study have pointed out that the results can be interpreted to support the efficacy of tapping, because the “sham” points selected by the investigators were in some cases actual acupuncture points (Pasahow, 2010; Church, 2013a). It is also noteworthy that all three tapping groups improved, while the non-tapping group did not.

A Canadian survey asked students about their recommendations for reducing stress and anxiety while enhancing coping skills (Ledger, 2019). It found that 67% of students recommended that EFT be taught in schools; 63% indicated they could benefit from learning EFT in smaller groups, and 33% indicated they would be interested in having one-on-one assistance from a counselor using EFT. Gaesser (2020) recommends formal training in EFT for stress and anxiety management for students and staff in school settings.

Since the publication of the anxiety meta-analysis (Clond, 2016), six RCTs comparing EFT to CBT have been performed, as was called for at the conclusion of the meta-analysis. Overall, these demonstrate that EFT and CBT have similar treatment effect sizes for anxiety. The number of sessions required for successful remediation of anxiety with EFT is small. When a subsequent meta-analysis including the new studies is performed, a statistical comparison between CBT and EFT will be available. Taken as a whole, these findings provide empirical support for EFT as a primary treatment for anxiety.

#### Depression

In 2016, an independent team conducted a meta-analysis of studies performed from 2005 to 2015 that evaluated the use of EFT to alleviate depression (Nelms and Castel, 2016). They identified 20 studies: 12 RCTs with 398 participants and 8 outcome studies with 461 participants. Depressive symptoms were compared at three different intervals: postintervention, follow-up in < 90 days, and follow-up in more than 90 days. Like Clond (2016), they noted that relatively few studies compared EFT to other established treatments. They came to the following conclusions: EFT was more efficacious than DB and supportive interview (SI) in posttest measurements (*p* = 0.06 vs. DB, *p* < 0.001 vs. SI) and sleep hygiene education (SHE) at follow-up (*p* = 0.036). No significant treatment effect difference between EFT and EMDR was found. EFT was superior to treatment as usual (TAU) and efficacious in treatment time frames ranging from one to 10 sessions. The mean of symptom reductions across all studies was −41%. Cohen's *d* across all studies was 1.31, indicating a large treatment effect, with little difference between randomized controlled trials and uncontrolled outcome studies. Effect sizes at posttest, < 90 and >90 days were 1.31, 1.21, and 1.11, respectively, indicating durable maintenance of participant gains. Table 4 shows the RCTs included in the Nelms and Castel (2016) meta-analysis. Note that the columns do not match the format of the other tables because of the way the analysis was performed, assessing outcomes at different time points.

**Table 4:** Depression RCTs included in Nelms and Castel (2016) meta-analysis.

**S.R.No**.	**References**	**Population**	**Analytical sample size**	**Instrument**	**Pre vs. post % change (%)**	**Posttest effect size *d***	**Posttest effect size SE (*d*)**	**Pre and posttest *p*-value**	**Posttest effect size *p* (*d*)**
1	Brattberg (2008)	Fibromyalgia patients	330	HADS	−29	0.62	0.26	*p* = 0.02	0.02
2	Church et al. (2013)	Veterans with PTSD	49	SA-45	−58	8.02	0.78	*p* = 0.001	< 0.001
3	Church and Brooks (2014)	Veterans at risk of PTSD	18	SA-45	−47	3.11	0.68	*p* = 0.001	< 0.001
4	Church et al. (2012c)	Nonclinical subjects	28	SA-45	−49	1.12	0.29	*p* = 0.001	< 0.001
5	Chatwin et al. (2016)	Patients with MDD	96	SA-45	−23	0.28	0.22	*p* = 0.01	0.21
6	Karatzias et al. (2011)	NHS psychotherapy referrals	23	HADS	−28	0.69	0.39	*p* = 0.001	0.08
7	Lee et al. (2013)	Senior insomnia patients	10	GDS-K	−60	1.41	0.41	*p* = 0.005	< 0.001
8	Church et al. (2012a)	Psychology students	9	BDI	−74	7.57	1.29	*p* ≤ 0.05	< 0.001
9	Geronilla et al. (2016)	Veterans with PTSD	58	SA-45	−48	1.93	0.3	*p* = 0.001	< 0.001
10	Church and Nelms (2016)	Adults with frozen shoulder	16	SA-45	−44	0.88	0.37	*p* = 0.001	0.02
11	Church et al. (2018c)	Veterans	16	SA-45	−38	0.9	0.36	*p* = 0.001	0.01
12	Stapleton et al. (2013)	Overweight and obese adults	45	SA-45	−21	0.37	0.23	*p* = 0.001	< 0.001

Weighted effect size—RCTs = 1.8498. BDI, Beck Depression Inventory; GDS-K, Geriatric Depression Scale in Korea; HADS, Hospital and Anxiety Depression Scale; MDD, Major Depressive Disorder; NHS, National Health Service; SA-45, Symptom Assessment−45. Sr. No., systematic review number.

Since the depression meta-analysis (Nelms and Castel, 2016), eight RCTs have been published. Four of these compare EFT to CBT and one to PMR.

An early study comparing EFT to CBT for Major Depressive Disorder (Chatwin et al., 2016) and a replication of this study (Jasubhai and Mukundan, 2018) revealed that both treatment approaches produced significant reductions in depressive symptoms. In the former, the CBT group reported a significant reduction postintervention, but this was not maintained over time. The EFT group reported a delayed effect involving a significant reduction in symptoms at the 3- and 6-month follow-ups only. Examination of the individual cases revealed clinically significant improvements in anxiety across both interventions. In the replication, EFT treatment produced marked improvement in depression symptoms after three sessions. EFT showed significant results within a month, compared to 8 weeks for CBT (Jasubhai and Mukundan, 2018).

In a Korean study comparing EFT and PMR, both EFT (*n* = 15) and PMR (*n* = 16) improved depression, with scores dropping further in the EFT group (−32.11 vs. −18.68%; Kwak et al., 2020). An Iranian study of the effect of EFT on depression in postmenopausal women (Mehdipour et al., 2021) showed that mean scores reduced in comparison to the control group *(p* = 0.001). At the end of 8 weeks, 63.4% of participants in the intervention group and 34.15% of controls were below the diagnostic threshold for depression (*p* < 0.001). The authors recommended using EFT in public health centers for postmenopausal women.

Secondary psychological outcomes, including depression, were evaluated in a trial comparing EFT to CBT in the treatment of food cravings (Stapleton et al., 2017). For EFT, preintervention to postintervention measured *p* = 0.017 with this improvement maintained at 6- and 12-month follow-ups (*p* = 0.016 and *p* = 0.116, respectively). There was no significant difference between groups at 12-month follow-up, indicating that depressive symptomatology for both EFT and CBT had reduced to the level of a nonclinical control group.

Several weight loss studies have delivered the EFT intervention online. In a 2-year follow-up to a trial for food cravings, the EFT group showed reduced depression (−12.3%) as a secondary outcome (Stapleton et al., 2019b). Pairwise comparisons revealed that symptoms decreased significantly from pre to posttest, from pre to 6-month follow-up (*p* < 0.001), and from pre to 12 months (*p* = 0.001). The chronic pain study mentioned above (Stapleton, 2022) showed significant reduction in depression from pre to post as well as pre to 6 months (*p* < 0.001).

After six sessions in a veterans' RCT (Church, 2014), there was a significant reduction in depression (*p* < 0.0001) and these gains were maintained at follow-up, after 6 months (*p* < 0.0001).

The study of Congolese survivors of sexual violence by Nemiro and Papworth (2015) described earlier used a general measure of mental health and did not report the 15 items of its depression component separately. It found similar effects for EFT and CBT.

Table 5 summarizes RCTs published since the Nelms and Castel (2016) meta-analysis.

**Table 5:** Depression RCTs published since the Nelms and Castel (2016) meta-analysis.

**S.R.No**.	**References**	**Population**	**Instrument**	**EFT (*n*) and sessions**	**Control/s (*n*) and sessions**	***p***
1	Kwak et al. (2020)	31 Hwabyung patients	THS, VAS-HS, BDI, STAI, STAXI	(*n* = 15) 4 group sessions	PMR (*n* = 16) 4 group sessions	*p < * 0.05
2	Jasubhai and Mukundan (2018)	10 community members with MDD	DASS-21, BDI-2	(*n* = 10) 8 sessions	1. CBT (*n* = 10) 8 sessions 2. Control group (*n* = 57)	Pre and post EFT DASS-21 anxiety: *p* = 0.02 BDI-2: *p* = 0.008
3	Chatwin et al. (2016)	10 community members with MDD	BDI-2, DASS-21	(*n* = 6) 1 session/week for 8 weeks	CBT (*n* = 4): 1 session/week for 8 weeks Community sample (*n* = 57)	*p* = 0.871 (same as CBT) *p* = 0.005 (compared to Community)
4	Mehdipour et al. (2021)	88 menopausal women	BDI-2	(*n* = 44) Self-participatory EFT 1/day for 8 weeks	Sham therapy (*n* = 44) Self-participatory sham acupressure points 1/day for 8 weeks	*p < * 0.001
5	Stapleton et al. (2019c)	314 people with food cravings and obesity	PHQ	(*n* = 145, at end of 12 months) 1 online session/week for 8 weeks	Wait-list control group (*n* = 137) NT	Anxiety score after 12 months postintervention *p < * 0.001
6	Stapleton et al. (2017)	83 overweight or obese adults	PHQ	(*n* = 51) 1 session/week for 8 weeks	CBT (*n* = 34) CBT: 1 session/week for 8 weeks	Both EFT and CBT at postintervention *p* = 0.097
7	Church (2014)	59 veterans with PTSD	SA-45	(*n* = 30) 6 sessions	WL (*n* = 29) NT	*p* ≤ 0.0001 (at both pretest and 6 month follow-up)
7	Nemiro and Papworth (2015)	50 female survivors of gender violence	HTQ, HSC-25	(*n* = 25) Two 2.5-h group treatment/week for 4 weeks	CBT (*n* = 25) 2 group treatments/week for 4 weeks	Both treatments had the same effect
8	Stapleton and Stewart (2020)	83 (49 for EFT) in-person and 314 online participants with food cravings	PHQ	In-person (*n* = 49) One 2 h session/week for 8 weeks	Online EFT (*n* = 314) Eight 2-h sessions split into 65 online modules designed to be accessed over 8 weeks	*p < * 0.001
10	Stapleton (2022)	168 chronic pain patients	PHQ	(*n* = 91) 6-week live facilitator led (*n* = 90) Self-paced online program	WL for live group (*n* = 45) WL for self paced (*n* = 50) WL group was later given EFT	Pre to post *p* < 0.001 Pre to 6-month *p* < 0.001

BDI, Beck Depression Inventory; BDI-2, Beck Depression Inventory, second edition; CBT, Cognitive Behavior Therapy; DASS-21, Depression, Anxiety, and Stress Scale−21; HSC-25, Hopkins Symptom Checklist−25; HTQ, Harvard Trauma Questionnaire; MDD, Major Depressive Disorder; PHQ, Patient Health Questionnaire; PMR, Progressive Muscular Relaxation; STAI, State-Trait Anxiety Inventory; STAXI, State-Trait Anger Expression Inventory; THS, The Hwabyung Scale; VAS-HS, Visual Analog Scale of Hwabyung Symptoms. Sr. No., systematic review number.

A noteworthy conclusion of the depression meta-analysis (Nelms and Castel, 2016) was that participant outcomes after EFT treatment were “equal or superior” to TAU and other active treatment controls. The posttest effect size for EFT (*d* = 1.31) was “larger than that measured in meta-analyses of antidepressant drug trials and psychotherapy studies.” The authors further noted that “EFT produced large treatment effects whether delivered in group or individual format, and participants maintained their gains over time” (p. 416). EFT may thus be regarded as a robust evidence-based treatment for depression.

#### Phobias

Three RCTs have been conducted for specific phobias such as fear of spiders, small animals, or heights. They are summarized in Table 6. The earliest study showed that the Clinical EFT protocol, including the tapping component, was more successful at reducing anxiety associated with a specific phobia (*p* < 0.005) than a control protocol that replaced tapping and EFT's cognitive reframing statement with DB (Wells et al., 2003).

**Table 6:** Phobia RCTs.

**S.R.No**.	**References**	**Population**	**Instruments**	**EFT (*n*) and sessions**	**Control(s) (*n*) and sessions**	***p***
1	Wells et al. (2003)	35 participants with specific phobia	FQ, DSM-4	(*n* = 18) One 45-min session	DB (*n* = 17) One 30-min session	*p < * 0.005
2	Baker and Siegel (2010)	31 participants with specific phobia	FQ, DSM-4	(*n* = 11) One 45-min session	1. NT (*n* = 10) 2. Interview (*n* = 10) 1 interview session	*p* = 0.136 *p* = 0.102
3	Salas et al. (2011)	22 students with specific phobia	BAT, SUD, BAI	(*n* = 11) Two rounds of EFT tapping, 5 min each	DB (*n* = 11) One session with five 2-min round of DB (*n* = 17)	Phobia related anxiety BAI: *p* = 0.042 SUD: *p* = 0.002 Ability to approach feared stimulus BAT: *p* = 0.046

BAI, Beck Anxiety Inventory; BAT, Behavioral Approach Test; DB, Diaphragmatic Breathing; DSM-4, Diagnostic and Statistical Manual of Mental Disorders, 4th edition; FQ, Fear Questionnaire; NT, no treatment; SUD, Subjective Units of Distress. Sr. No., systematic review number.

A replication and extension by Baker and Siegel (2010) assessed whether such findings reflected (a) nonspecific factors common to many forms of psychotherapy; (b) a methodological artifact such as regression to the mean, fatigue, or the passage of time; and/or (c) therapeutic ingredients specific to EFT. Using a carefully prepared design, it found that the effects noted in Wells et al. (2003) were due to EFT and not to experimental artifacts.

A second study designed explicitly as a partial replication of Wells et al. (2003) also used DB as a control intervention (Salas et al., 2011). It found that EFT significantly reduced specific phobia-related anxiety (*p* = 0.042) and the ability of participants to approach the feared stimulus (*p* = 0.046).

The two independent replications of the initial phobia study by Wells et al. (2003) provides strong empirical support for EFT's efficacy for phobias.

König et al. (2019) performed an RCT comparing EFT to PMR and measuring brain activity for responses during fear stimuli using EEG. The study authors posit that if the emotional experience of fear is elicited by anger rather than fearful stimuli, it is plausible that tapping alters the processing of these stimuli, as their reframing is an important part of the tapping intervention. Consequently, the Late Positive Potential (an important component in Explicit Recognition Memory) might decrease.

#### PTSD

An independent meta-analysis using the APA criteria for quality control identified seven RCTs (Sebastian and Nelms, 2017). It concluded that EFT is efficacious and reliable as a treatment for PTSD in time frames ranging from four to 10 sessions. The effect size resulting from treatment was extremely large, with *d* = 2.98. No treatment effect difference was found in studies comparing EFT to other evidence-based therapies such as EMDR and CBT, though like the anxiety and depression meta-analyses, a limited number of studies (one for each of these two methods) was available for comparison. The authors concluded that EFT is safe and effective, can be used as a self-help practice, and is applicable to heterogeneous populations. Table 7 shows the RCTs included in the Sebastian and Nelms meta-analysis.

**Table 7:** PTSD RCTs included in Sebastian and Nelms (2017) meta-analysis.

**S.R.No**.	**References**	**Population**	**Instruments**	**EFT (*n*) and sessions**	**Control(s) (*n*) and sessions**	**Cohen's *d* (95% CI) and/or *p*-value**
1	Karatzias et al. (2011)	46 NHS patients with PTSD	CAPS, PCL-C, HADS	(*n* = 23) Four 1-h sessions/individual	EMDR (*n* = 23) Four 1-h sessions/individual	PCL: 1.08 (0.38–1.73)
						Anxiety: 1.11 (−0.41–1.77)
						Depression:0.69 (−0.02–1.32)
2	Church et al. (2012b)	16 institutionalized teenage boys	SUD, IES	(*n* = 8) 1 session	WL (*n* = 8) NT	Intrusive memories −3.95 (2.26–5.63)
						Avoidance: 6.89 (4.31–9.47)
						IES total: 8.07 (5.11–11.03)
3	Church et al. (2013)	59 veterans with PTSD	SA-45, PCL-M	(*n* = 30) Six 1-h sessions	WL (SOC/NT) (*n* = 29)	PCL: 1.93 (1.28–2.58) Psychological distress (*p < * 0.0012)
						Anxiety: 1.36 (0.77–1.95) PTSD symptom level *p < * 0.0001
						Depression: 1.76 (1.13–2.39)
4	Geronilla et al. (2016)	58 veterans with PTSD	SA-45, ISI, PCL-M	Six 1-h sessions (*n* = 32)	WL (*n* = 26) TAU	PCL: 3.06 (2.30–3.82)
						Anxiety: 1.55 (0.96–2.14)
						Depression: 1.65 (1.06–2.25)
5	Church et al. (2018c)	16 veterans with PTSD	SA-45, HADS, ISI, BPI, PCL-M	(*n* = 8) Ten 1-h sessions	WL (*n* = 8) TAU	PCL: 2.18 (1.25–2.99) PTSD symptoms *p < * 0.00001
						Anxiety: 0.78 (0.04–1.47) Significant differential expression of six genes was found (*p < * 0.05)
						Depression:0.89 (0.15–1.60)
6	Church et al. (2016)	21 veterans at risk of PTSD	SA-45, ISI, PCL-M	(*n* = 12) Six 1-h sessions	WL(*n* = 9) TAU	PCL: 6.63 (4.44–8.81)
						Anxiety: 3.64 (2.24–5.04)
						Depression: 4.32 (2.76–5.89)
7	Nemiro and Papworth (2015)	50 female survivors of gender violence	HTQ, HSCL	(*n* = 25) Two 2.5-h group sessions/week for 4 weeks	CBT (*n* = 25) 2 group treatments/week for 4 weeks for 4 weeks	HSCL: 1.26 (0.61–1.87) HTQ: 2.29 (1.51–2.99)

BPI, Brief Pain Inventory; CAPS-5, Clinician-Administered PTSD Scale for DSM-5; CBT, Cognitive Behavior Therapy; EMDR, Eye Movement Desensitization and Reprocessing; HADS, Hospital and Anxiety Depression Scale; HSCL, Hopkins Symptom Checklist; HTQ, Harvard Trauma Questionnaire; IES, Impact of Event Scale; ISI, Insomnia Severity Index; NHS, National Health Service; NT, no treatment; PCL-C, PTSD Checklist–Civilian; PCL-M, PTSD Checklist–Military; SA-45, Symptom Assessment−45; SUD, Subjective Units of Distress; TAU, treatment as usual; WL, wait-list. Sr. No., systematic review number.

More recently, a second independent team undertook a systematic review and network meta-analyses of psychological and psychosocial interventions for children and young people with PTSD (Mavranezouli et al., 2019) and for adults (Mavranezouli et al., 2020). The former included 32 trials of 17 interventions involving 2,260 participants and the latter included 90 trials, 6,560 individuals, and 22 interventions. The study included interventions such as trauma-focused CBT (TF-CBT), EMDR, and talk therapy. In the 2019 study, EFT proved to be one of the two most effective therapies in reducing PTSD symptoms at the treatment endpoint, while it demonstrated the second best results with Standard Mean Difference (SMD) = −1.69 in the 2020 study. The investigators noted the positive evidence for EFT. However, they also considered its limited evidence base beyond the treatment endpoint and recommended EMDR and TF-CBT as the two therapies with the greatest evidence bases.

The earlier meta-analysis by Sebastian and Nelms (2017) included a study of the effects of EFT treatment on veterans with PTSD (Church et al., 2013), as well as a replication of this study (Geronilla et al., 2016). In the initial study (*N* = 59), after six treatment sessions, and a 6 month follow-up period, 90% of the participants no longer qualified for a clinical diagnosis of PTSD (Church et al., 2013). The replication (*N* = 58) found similar treatment effects (Geronilla et al., 2016).

The outcomes of the two studies were remarkably similar. After treatment, 90% of participants in the initial study and 96% of those in the replication had dropped below the diagnostic threshold for PTSD. The mean scores on the PTSD assessment used dropped from 64 to 37 in the initial study and from 65 to 34 in the replication. Significant reductions in other forms of psychological distress were significant in both the first trial (*p* = 0.0012) and the second (*p* = 0.001). On long-term follow-up, 86% of participants in the initial study and 95% in the replication no longer met the PTSD diagnostic criteria. The meta-analysis also included a study of veterans at risk for PTSD because of heightened symptom levels. Performed by the same research team as the first PTSD study, it used methodology identical to the above two trials. Symptom declines were similar (Church et al., 2016). Two replications thus confirmed the results of the initial study (Church et al., 2013).

The data from the initial PTSD trial (Church et al., 2013) were reanalyzed to examine the efficacy of EFT when delivered by life coaches vs. licensed mental health professionals (Stein and Brooks, 2011). It found larger reductions in symptoms in veterans treated by licensed practitioners, though the difference did not rise to the level of statistical significance. A second reanalysis compared phone to in-person treatment (Hartung and Stein, 2012). While in-person treatment was significantly superior, nonetheless 67% of those treated by phone no longer met the diagnostic criteria for PTSD at a 6-month follow-up. These analyses indicate the utility of EFT when delivered over the telephone and by practitioners with basic levels of training.

The meta-analysis also included an extension of these studies. While analyzing the psychological symptoms of veterans, it also measured the physiological effects of treatment. Gene expression as well as other physiological markers were examined. EFT was found to produce epigenetic effects, with upregulation of genes associated with improved immunity and the control of inflammation (Church et al., 2018c). Though it had not yet been published, data from this study had been reported at the time of the meta-analysis, so it was included. It thus appears in Table 7 rather than Table 8, which shows the PTSD RCTs published since the meta-analysis.

**Table 8:** PTSD RCT published since Sebastian and Nelms (2017) meta-analysis.

**S.R.No**.	**References**	**Population (verification tool)**	**Instrument**	**EFT sessions**	**Control(s)**	***p***
1	Al-Hadethe et al. (2015)	60 male Iraqi students with PTSD	Scale of Posttraumatic Stress Syndromes	4 sessions (*n* = 20)	Narrative Exposure Therapy (*n* = 20) 4 sessions	Pretest and posttest from T1 to T2 *p* > 0.05
					Control group (*n* = 20) NT	

DSM-4, Diagnostic and Statistical Manual of Mental Disorders, 4th edition; NT, no treatment. Sr. No., systematic review number.

A second RCT of EFT for PTSD has been reported since the date of the Sebastian and Nelms (2017) meta-analysis and appears in Table 8. In a comparison of EFT and Narrative Exposure Therapy (NET), both EFT and NET demonstrated efficacy (Al-Hadethe et al., 2015). Participants were 60 secondary school students aged 16–19 years who met the criteria for PTSD defined in the *Diagnostic and Statistical Manual of Mental Disorders*, fourth edition (DSM-IV). Between pre and posttest, both treatments produced improvements in variables such as anxiety, reexperience, and avoidance behavior. However, EFT also led to statistically significant improvements in depression and hyperarousal. The effect sizes for EFT were greater than for NET. While participant gains were durable in the EFT group on 3-, 6-, and 12-month follow-up, they were unstable for NET. Table 8 does not include the Babamahmoodi et al. (2015) RCT with veterans, because while anxiety and other mental health issues were measured, PTSD was not.

There are two outcome studies that are not RCTs that are worth examining for their clinical implications. At Fort Hood, the largest military base in the US, EFT as well as other complementary therapies were available to traumatized warriors through the Warrior Combat Stress program for 7 years, 2008–2015. Treatment outcomes were analyzed for 764 service members who attended a 3-week program between 2008 and 2013 (Libretto et al., 2015). The investigators identified significant declines in PTSD, anxiety, depression, and pain (all *p* < 0.001).

The second study examined PTSD symptoms in veterans and their spouses attending one of six 7-day retreats (*n* = 218). The investigators hypothesized that social support could provide a useful adjunct to EFT and other complementary therapies. At the start of the retreats, 83% of veterans and 29% of spouses met the PTSD diagnostic criteria. By the end of the 7 days, only 28% of veterans and 4% of spouses were still in the clinical range (Church and Brooks, 2014). Follow-up results were similar, with spousal symptom levels dropping even further. When each of the six retreats was analyzed independently, as though it were a small study in and of itself, the downward slope of symptoms was similar to that observed in the aggregated statistics.

Other studies find EFT effective for PTSD in a variety of populations. These include motor vehicle accident survivors (Swingle et al., 2004), business owners (Church and House, 2018), victims of sexual assault (Anderson et al., 2019), Haitian earthquake survivors (Gurret et al., 2012), Congolese gender violence victims (Nemiro and Papworth, 2015), adolescents (Church et al., 2012b), civilian survivors of war (Boath et al., 2014b), and earthquake survivors (Rahmi, 2012).

In a study gathering survey responses from 448 EFT practitioners who had experience treating clients with PTSD, 65% stated that more than 60% of PTSD clients are fully rehabilitated, and 89% of practitioners stated that fewer than 10% of clients make little or no progress (Church et al., 2017). The authors of the meta-analysis concluded that EFT “can be used both on a self-help basis and as a primary evidence-based treatment for PTSD” (p. 16). The evidence accumulated since the publication of the meta-analysis augments the body of literature supporting EFT's status as an empirically based treatment for a wide variety of traumatized populations.

### Physiological issues—Somatization, pain, physical symptoms, weight loss, insomnia, gene expression, autoimmune conditions, hormones, and cravings

The first decade after the turn of the current century witnessed extensive measurement of the psychological improvements produced by EFT. The subsequent decade produced increased investigation of EFT's physiological dimensions. The results of these studies are presented below. A meta-analysis is available for only one, somatic symptoms, therefore trials lower down the hierarchy of evidence are presented for the others.

#### Somatic symptoms

A meta-analysis examined EFT's effect on somatic symptoms (Stapleton et al., 2021). Using the Cochrane Collaborative procedure and filtering studies through the APA criteria for quality control, eight RCTs (*n* = 640) were identified. Upon analysis, the effect size pre-post EFT was found to be *d* = −1.09, indicating a large treatment effect (95% CI, −1.217 to −0.964, *p* < 0.001). These results are presented in Table 9. The essential characteristics of those studies not qualifying for the meta-analysis appear in Table 10.

**Table 9:** Somatization RCTs included in the Stapleton et al. (2021) meta-analysis.

**S.R.No**.	**References**	**Population**	**Instrument**	**Control(s)**	**Results type**	Cohen's d^**^	***p***
1	Stapleton et al. (2020b)	53 nonclinical adults	SA-45	Psychoeducation (*n* = 17) One 60-min session NT (*n* = 17)	Pre/post EFT^***^	−0.16	*p* = 0.60
					Post EFT vs. post control	0.11	*p* = 0.72
2	Stapleton et al. (2019c)	282 overweight adults	PHQ	WL (*n* = 137)	Pre/post EFT	−0.48	*p < * 0.0001^*^
					Post EFT vs. post control	0.46	*p* = 0.0002^*^
					Post to 6-month follow-up	−5.18	*p < * 0.0001^*^
3	Stapleton et al. (2017)	83 overweight adults	PHQ	CBT (*n* = 34) 1 session/week for 8 weeks	Pre/post EFT	−0.36	*p* = 0.068
					Post EFT vs. post control	0.35	*p* = 0.10
					Post to 6-month follow-up	0.3	*p* = 0.14
4	Geronilla et al. (2016)	58 veterans with PTSD	SA-45	Standard care (*n* = 26)	Pre/post EFT	−1.5	*p < * 0.0001^*^
					Post EFT vs. post control	1.18	*p* = 0.0001^*^
					Post to 6-month follow-up	0.22	*p* = 0.41
5	Church et al. (2018c)	16 veterans with PTSD	SA-45	Standard care (*n* = 8)	Pre/post EFT	−0.055	*p* = 0.628
					Post to 6-month follow-up	0.11	*p* = 0.89
6	Church et al. (2016)	21 veterans at risk of PTSD	SA-45	Standard care (*n* = 9)	Pre/post EFT	−3.23	*p < * 0.0001^*^
					Post EFT vs. post control	2.93	*p < * 0.0001^*^
					Post to 6-month follow-up	1.54	*p* = 0.001^*^
7	Church et al. (2013)	59 veterans with PTSD	SA-45	Standard care (*n* = 29)	Pre/post EFT	−6.71	*p < * 0.0001^*^
					Post EFT vs. post control	5.32	*p < * 0.0001^*^
					Post to 6-month follow-up	0.84	*p* = 0.002^*^
8	Church et al. (2012c)	83 nonclinical adults	SA-45	Psychoeducation (*n* = 28) One 50-min session	Pre/post EFT	−1.2	*p < * 0.0001^*^
					Post EFT vs. post control	1.15	*p* = 0.0001^*^

* Significance *p* < 0.05.

** Cohen's *d*: small, *d* = 0.2, medium, *d* = 0.5, and large, *d* = 0.8.

*** follow-up was 1 h only, therefore no data available. CBT, Cognitive Behavior Therapy; NT, no treatment; PHQ, Patient Health Questionnaire; SA-45, Symptom Assessment−45; WL, wait-list. Sr. No., systematic review number.

**Table 10:** RCTs of EFT studies on somatic symptoms not included in the Stapleton et al. (2021) meta-analysis.

**S.R.No**.	**References**	**Population**	**Instrument**	**EFT (*n*) and sessions**	**Control/s (*n*) and sessions**	***p* (EFT)**
1	Tack et al. (2021)	121 cancer survivors with cognitive impairment	CFQ	(*n* = 59) 8 weeks EFT + 8 weeks observation	WL (*n* = 62) Received EFT after 8 weeks	*p < * 0.01
2	Cici and Özkan (2021)	162 patients with lumbar disc herniation	PIF, LFF, SUD, STAI-S	(*n* = 54) 30 min	Music group (*n* = 54) 30 min Control group (*n* = 54) NT	*p < * 0.05
3	Babamahmoodi et al. (2015)	28 chemically pulmonary injured veterans	GHQ, SGRQ	(*n* = 14) 8-week group session	WL control group (*n* = 14) No treatment but received EFT program after second evaluation	Somatic symptoms: *p* = 0.02 Frequency and severity of respiratory symptoms: *p < * 0.001
4	Ghaderi et al. (2021)	50 women with multiple sclerosis	FSS	(*n* = 25) 2 sessions/week for 4 weeks	Placebo tapping on non-acupuncture points (*n* = 25) 2 sessions/wk for 4 weeks	Fatigue severity after 4 weeks *p < * 0.001

CFQ, Cognitive Failures Questionnaire; FSS, Fatigue Severity Scale; GHQ, General Health Questionnaire; LFF, Life Findings Form; PIF, Patient Information Form; SGRQ, Saint George Respiratory Questionnaires; STAI-S, State and Trait Anxiety Scores; SUD, Subjective Units of Distress; WL, wait-list. Sr. No., systematic review number.

The meta-analysis used the somatization scale employed in its component studies. Other trials have examined a variety of physiological markers other than somatization. A 2019 outcome study sought to elucidate EFT's mechanisms of action across the central nervous system (CNS) by measuring heart rate variability (HRV) and heart coherence (HC); the circulatory system using resting heart rate (RHR) and blood pressure (BP); the endocrine system using cortisol; and the immune system using salivary immunoglobulin A (SigA). Posttest, significant declines were found in pain (−57%), and cravings (−74%), all *p* < 0.00. Happiness increased (+31%, *p* = 0.000), as did SigA (+113%, *p* = 0.017). Significant improvements were found in RHR (−8%, *p* = 0.001), cortisol (−37%, *p* < 0.000), systolic BP (−6%, *p* = 0.001), and diastolic BP (−8%, *p* < 0.000). Positive trends were observed for HRV and HC and gains were maintained on follow-up, indicating EFT results in positive health effects as well as increased mental wellbeing (Bach et al., 2019).

A key early RCT examined EFT's effects on the stress hormone cortisol (Church et al., 2012c). It found that in a single session, psychological symptoms dropped twice as much in an EFT group as in groups either resting or engaging in a talk therapy session. Cortisol dropped significantly more. A direct replication of this study was undertaken (Stapleton et al., 2020b). The EFT group experienced a decrease in cortisol greater than in the original study (−43.24%, *p* < 0.05). This was superior to that of a control psychoeducation group (−19.67%), as well as a no treatment (NT) group (2.02%).

Studies have also shown that EFT is associated with epigenetic effects. A PTSD study referenced previously (Church et al., 2018c) found regulation of six genes associated with inflammation and immunity. A pilot study comparing an hour-long EFT session with placebo in four nonclinical participants found differential expression in 72 genes (Maharaj, 2016). These included genes associated with the suppression of cancer tumors, protection against ultraviolet radiation, regulation of type 2 diabetes insulin resistance, immunity from opportunistic infections, antiviral activity, synaptic connectivity between neurons, synthesis of both red and white blood cells, enhancement of male fertility, building white matter in the brain, metabolic regulation, neural plasticity, reinforcement of cell membranes, and the reduction of oxidative stress.

As part of the meta-analysis (Stapleton et al., 2021), the investigators also examined the studies of EFT for physical conditions other than somatization but found the heterogeneity among them to be too large to combine for analysis. We thus must rely on the individual studies to present an evaluation of EFT's physiological effects.

An RCT compared cognitive function in cancer survivors. The experimental group received EFT for 16 weeks and was compared to a WL group. After treatment, less than half those in the EFT group scored positive for cognitive impairment compared to controls (4.8 vs. 87.3% respectively; *p* < 0.01). Linear mixed model analyses indicated a statistically significant reduction in cognitive impairment scores and also in distress, depressive symptoms, and fatigue. Quality of life improved (Tack et al., 2021).

In an RCT conducted with patients hospitalized for surgical treatment of lumbar disc herniation, EFT and MT were determined to remediate participants' state anxiety and subjective discomfort (*p* < 0.001). EFT significantly reduced pulse and respiratory rates, as well as systolic blood pressure, while MT significantly lowered both diastolic and systolic blood pressure (*p* < 0.05). Further analyses showed that EFT was more effective for state anxiety and reducing respiratory rate than MT (Cici and Özkan, 2021).

Ghaderi et al. (2021) conducted an RCT measuring effect of EFT on the severity of fatigue among women with multiple sclerosis (MS). The investigators identified a significant decrease in symptoms, both immediately after treatment, and 4 weeks after the intervention (*p* < 0.001). An RCT was conducted with a population of chemically pulmonary injured veterans (Babamahmoodi et al., 2015). Veterans injured with chemical weapons encounter many stressors such as chronic respiratory problems, as well as war-induced psychological and physical problems that affect their health, immunity, and quality of life. In this population, EFT improved mental health (*p* = 0.000) and health-related quality of life (*p* = 0.001). Treatment was associated with decreased somatic symptoms (*p* = 0.02), anxiety/insomnia (*p* < 0.001), social dysfunction (*p* < 0.001), and frequency and severity of respiratory symptoms (*p* < 0.001).

The authors of the meta-analysis concluded: “Clinical EFT is effective in reducing somatic symptoms in a variety of populations and settings. As a fast-acting, patient-applied, non-pharmacological and evidence-based method, EFT is recommended in primary settings for somatoform disorders” (Stapleton et al., 2021, p. 1). The results of the RCTs not included in the meta-analysis, as well as the uncontrolled outcome studies, support this conclusion. The literature demonstrates that EFT is an effective, evidence-based primary intervention for treating somatic symptoms.

Many physical symptoms have responded favorably to EFT treatment. A study comparing EFT to DB among participants with frozen shoulders (Church and Nelms, 2016) showed that both groups improved after treatment, but only the EFT group maintained their gains over time (47% reduction in symptoms, *p* < 0.001). Large treatment effects were found, with a Cohen's *d* = 0.9 for anxiety and pain. Reductions in psychological distress were associated with reduced pain as well as with improved range of motion.

In women suffering from fibromyalgia, pain catastrophizing measures such as rumination (*p* < 0.001), magnification (*p* = 0.006), and helplessness (*p* < 0.001) were significantly reduced and their activity level significantly increased (*p* = 0.001) posttest (Brattberg, 2008). A study of patients with tension headaches performed at the Red Cross Hospital in Athens found that the frequency and intensity of their headaches dropped by more than half after EFT (*p* < 0.001), and other physical symptoms improved (Bougea et al., 2013). In a study of 39 business executives using EFT as a group (Church and David, 2019), pain was reduced by 41% and cravings relating to food and drink reduced by 50%.

Apart from the previous studies, several other studies on pain (Church and Brooks, 2010; Church, 2014; Ortner et al., 2014), obesity (Stapleton et al., 2012, 2016a), traumatic brain injury (Church and Palmer-Hoffman, 2014), and seizure disorders (Swingle, 2010) have demonstrated the ability of EFT to treat a disparate range of physiological conditions.

#### Pain

Stress and pain are related. When psychological stress is reduced by treatment, reductions in physical pain are often noted. Many EFT studies have assessed pain as a primary or secondary outcome measure. These are summarized in Table 11.

**Table 11:** RCTs measuring pain as a primary or secondary outcome.

**S.R.No**.	**References**	**Population**	**Instruments**	**EFT (*n*) and sessions**	**Control/s (*n*) and sessions**	***p* or *d* or %**
1	Church and Nelms (2016)	47 participants with frozen shoulder	MPRS	(*n* = 16) 1 individual 30-min Clinical EFT session	DB (*n* = 18) 1 individual 30-min session WL control group (*n* = 13)	Pain EFT: posttest *p* = 0.003 Follow-up *p* = 0.004 Pain DB: posttest *p* = 0.004 Follow-up *p* = 0.018
2	Bougea et al. (2013)	35 tension headache sufferers	SFQ-36 (One of the variables in this questionnaire is body pain)	(*n* = 19) Twice a day for 2 months	Standard care (*n* = 16) TAU	*p* = 0.008
3	Brattberg (2008)	62 fibromyalgia patients	SFQ-36 (One of the variables in this questionnaire is body pain), PCS, CPAQ	(*n* = 26) 8 weeks	WL (*n* = 36)	Body pain *p* = 0.20 Rumination (*p* < 0.001), Magnification (*p* = 0.006), and Helplessness due to pain (*p* < 0.001)
4	Stapleton (2022)	168 chronic pain patients	PCS, CPAQ	(*n* = 91) 6-week live facilitator led Self-paced online program (*n* = 90)	WL for live group (*n* = 45) WL for self paced (*n* = 50) WL group was later given EFT	Pain severity and Pain Interference pre to post *p* < 0.001 Pre to 6-month *p < * 0.001
5	Church (2014)	59 veterans with PTSD	MPRS	(*n* = 30) 6 sessions	WL (*n* = 29) NT	−41%, *p* < 0.0001
6	Bakir et al. (2021)	50 nursing students with PMSS	PMSS	(*n* = 25) Self EFT participation for 3 menstrual cycles	WL (*n* = 25) NT	*p < * 0.05
7	Geronilla et al. (2016)	58 veterans with PTSD	MPRS	(*n* = 32) 6 sessions	WL (*n* = 26) TAU	*p* < 0.001
8	Church et al. (2018c)	16 veterans with PTSD	BPI-PS, BPI-PI	(*n* = 8) 1 session/week for 10 weeks	WL (*n* = 8) TAU	BPI-PS at posttest *p* = 0.025 and 6 mo *p* = 0.188 BPI-PI at posttest *p =* 0.009 and 6 mo *p* = 1
9	Church et al. (2016)	21 veterans at risk of PTSD	MPRS	(*n* = 12) 1-h sessions/week for 6 weeks	WL (*n* = 9) TAU	*p* = 0.835

BPI-PI, Brief Pain Inventory–Pain Interference; BPI-PS, Brief Pain Inventory–Pain Scale; CPAQ, Chronic Pain Acceptance Questionnaire; DB, Diaphragmatic Breathing; GHQ, General Health Questionnaire; GSES, General Self-Efficacy Scale; GSI, Global Severity Index; HADS, Hospital and Anxiety Depression Scale; MHLCS, Multidimensional Health Locus of Control Scale; MPRS, Matheson Pain Rating Scale; NT, no treatment; PCS, Pain Catastrophizing Scale; PMSS, Premenstrual Syndrome Scale; PSS, Perceived Stress Scale; SA-45, Symptom Assessment−45; SFQ-36, Short-Form Questionnaire−36; SGRQ, Saint George Respiratory Questionnaires; SOC, standard of care; SUD, Subjective Units of Distress; TAU, treatment as usual; WL, wait-list. Sr. No., systematic review number.

Brattberg's 2008 RCT of women with fibromyalgia found significant reductions in three dimensions of pain using the Pain Catastrophizing Scale. These were rumination (*p* < 0.001), magnification (*p* = 0.006), and helplessness due to pain (*p* < 0.001). The same study reported increased levels of activity on the Chronic Pain Acceptance Questionnaire (*p* = 0.001).

Veterans were also found to experience significant drops in physical pain after EFT (*p* < 0.0001; Church, 2014), and when PTSD was remediated, symptoms of traumatic brain injury (TBI) reduced by 41% after three sessions (*p* < 0.0021; Church and Palmer-Hoffman, 2014). Participant gains were maintained on 3- and 6-month follow-up (*p* < 0.0006). The study of patients with frozen shoulder summarized above also found reductions in pain (Church and Nelms, 2016), as did the study of tension headache sufferers (Bougea et al., 2013).

An RCT for chronic pain (Stapleton, 2022) delivered EFT in two formats: self-paced (online) and facilitator-led (delivered live online due to COVID). Participants (*N* = 168) were randomly allocated to either option or WL. Pain was significantly less from baseline (pre) to 6-month follow-up (*p* < 0.001). A significant positive correlation was found between the ACE (adverse childhood experience) score and Pain Interference scores, somatic symptoms, anxiety symptoms, and depressive symptoms. As the ACE score increased, so did these other variables. Similarly, a negative correlation was found between the ACE score and Quality of Life (QoL). As the ACE score increased, QoL decreased. QoL improved (pre to 6 months, *p* < 0.001) as pain reduced.

An RCT of Turkish nursing students with premenstrual syndrome (PMS; Bakir et al., 2021) found improvements in depression, fatigue, nervousness, sleep-related changes, swelling, and other PMS symptoms (*p* < 0.05).

The Geronilla et al. (2016) replication study of Church et al. (2013) showed reduction in pain symptoms and this was maintained at 6-month follow-up (*p* < 0.001). In the Church et al. (2018c) study of the epigenetic results of Clinical EFT treatment, the severity of conditions commonly noted as sequelae to traumatic stress, including pain, declined, suggesting a general stress reduction effect (*p* = 0.009). However, in the study of subclinical veterans at risk of PTSD (Church et al., 2016), changes in pain were not significant *(p* = 0.835).

A number of uncontrolled studies merit mention due to their clinical significance. Pain was significantly reduced by 41% and cravings relating to food and drink dropped by 50% in an uncontrolled study of 39 business executives using EFT as a group during a daylong workshop (Church and David, 2019). A study of 216 health care workers identified a 68% reduction in physical pain (*p* < 0.001; Church and Brooks, 2010).

Ortner et al. (2014) observed significant improvements in pain severity, interference, life control, affective distress, and dysfunction, with pain catastrophizing dropping significantly over the course of a 3-day workshop (−42%, *p* < 0.001). Stapleton et al. (2016b) offered a brief intensive 4-h treatment protocol to participants in a persistent pain program and found a significant decrease in the severity (−12.04%, *p* = 0.044) and impact (−17.62%, *p* = 0.008) of pain.

The Scandinavian Association for the Study of Pain has shown evidence of the relationship between physical pain and anxiety and stress (Curtin and Norris, 2017). The research presented above provides clear evidence of EFT's ability to reduce pain reliably and quickly. It is effective when delivered in a variety of formats, in both clinical settings and as self-help, and in brief treatment time frames.

#### Physical symptoms

Studies show that EFT's effects are measurable in physical symptoms other than pain (see Table 12). This includes psychoimmunological factors, blood sugar, quality of life, perceived stress, cortisol salivary levels, seizures, and certain autoimmune conditions.

**Table 12:** RCTs assessing other physical symptoms.

**S.R.No**.	**References**	**Population**	**Instrument**	**EFT (n) and sessions**	**Control/s (*n*) and sessions**	**Results**
1	Babamahmoodi et al. (2015)	28 chemically pulmonary injured veterans	Immunological tests	(*n* = 14) Eight 90-min weekly group sessions and 2 times daily home practices	WL control group (*n* = 14)	1. Increased lymphocyte proliferation a. Con A (*p* = 0.001) b. PHA (*p* = 0.002)| c. Peripheral blood IL-17 (*p* = 0.006) 2. Reduced severity of respiratory symptoms (*p < * 0.001) 3. Decreased social dysfunction (*p < * 0.001),
2	Hajloo et al. (2014)	30 diabetic patients with high blood sugar and HbA1c>6	HbA1C	(*n* = 15) 12 sessions across 3 months	NT (*n* = 15)	Fob:7.24 > Fcr:4.22
3	Bougea et al. (2013)	35 patients with tension-type headache	MHLCS	(*n* = 19) Twice a day for 2 months	NT control group *n* = 16	Improved physical functioning (*p* = 0.005)
4	Brattberg (2008)	26 women with fibromyalgia	SF-36, HQ, GSE	(*n* = 26) 8 weeks *via* internet	WL (*n* = 36)	Role-physical: ability to manage daily life with physical impairment (*p* = 0.001)

Con A, concanavalin A; GSE, General Self-Efficacy scale; HbA1c, test measuring sugar level in hemoglobin A1c; HQ, Health Questionnaire; IL-17, interleukin 17; MHLCS, Multidimensional Health Locus of Control Scale; NT, no treatment; PHA, phytohemagglutinin; SF-36, Short-Form−36 questionnaire; WL, wait-list. Sr. No., systematic review number.

The Babamahmoodi et al. (2015) study mentioned in other sections indicated decreased social dysfunction (*p* < 0.001) and frequency and severity of respiratory symptoms (*p* < 0.001). This test also used the Lymphocyte Transformation Test (LTT), which measures lymphocyte proliferation in response to stimuli. The greater the proliferation, the more effective the immune response. Post EFT tests showed increased lymphocyte proliferation with nonspecific mitogens concanavalin A (Con A; *p* = 0.001), phytohemagglutinin (PHA; *p* = 0.002), and peripheral blood interleukin 17 (IL-17; *p* = 0.006).

In an investigation of EFT's effectiveness in diabetic patients' blood sugar control (Hajloo et al., 2014), 30 diabetic patients with high blood sugar and HbA1c > 6 were randomly classified into an EFT treatment group and non-treatment group. The study concluded that EFT is associated with a reduction of blood sugar levels (Fob:7.24>Fcr:4.22). Significant differences were found after EFT intervention in the Bougea et al. (2013) RCT with tension-type headache patients, particularly in physical functioning (*p* = 0.005), role limitations due to physical health (*p* = 0.001), energy/fatigue (*p* = 0.001), and general health (*p* = 0.002), except for social functioning (*p* = 0.082).

In the RCT with fibromyalgia patients (Brattberg, 2008), those who practiced EFT reported increased activity levels and their ability to manage daily life with physical impairment improved (*p* = 0.001) in comparison to the wait-list group.

Apart from the RCTs listed above, there are a few studies that provide useful clinical indicators. A study measuring the effect of EFT on psoriasis (Hodge and Jurgens, 2011) showed significant improvement in psychological, emotional, and physical symptoms. Assessments indicated improvements in psoriasis symptoms (−49.05%; *p* = 0.001) and functioning (−58.31%; *p* = 0.001) posttest, as well as a decrease in emotional distress (−41.56%, *p* = 0.002). In conjunction with the fibromyalgia and MS studies, this suggests that Clinical EFT can moderate the symptoms of autoimmune conditions.

A service evaluation assessed EFT for improving mood, menopausal symptoms, and fatigue in women with breast cancer receiving hormonal therapies (Baker and Hoffman, 2014). At both 6 and 12 weeks, statistically significant improvements were found in both mood (*p* = 0.005, *p* = 0.008) and fatigue (*p* = 0.008, *p* = 0.033). Improved results in seizure disorders (Swingle, 2010) as well as clinical case histories of dyslexia (McCallion, 2012) and TBI (Craig et al., 2009) are suggestive of a range of conditions for which EFT's stress-reduction ability can provide relief.

Hypotheses about why a psychological and energy treatment like EFT is effective for a heterogeneous group of physiological ailments range from the technique's capacity to rapidly reduce stress levels (Lane, 2009; Church et al., 2012c) to its postulated strengths in facilitating the adaptive processing of emotional information (Feinstein, 2015). However, EFT cannot yet be considered an evidence-based treatment for the physiological conditions listed above because of the small number of well-designed RCTs available and the lack of independent replications.

#### Insomnia

Six RCTs have examined the effect on insomnia after EFT treatment (see Table 13). The veterans' PTSD study referenced previously (Church et al., 2013) found a significant improvement in insomnia scores, with mean values dropping from the clinical to the subclinical range (*p* < 0.001). It also showed significant correlations (*p* < 0.03) between insomnia and anxiety (*r* = 0.40), depression (*r* = 0.41), interpersonal sensitivity (*r* = 0.29), and the Global Severity Index (GSI; *r* = 0.32). The replications of that study identified similar effects. The RCT of pulmonary-impaired Iranian veterans also identified decreased insomnia (*p* < 0.001; Babamahmoodi et al., 2015). Reduction of insomnia was noted in veterans at risk of PTSD (*p* = 0.004; Church et al., 2016) as well as in the epigenetic study described previously (*p* = 0.005; Church et al., 2018c).

**Table 13:** RCTs measuring insomnia as a primary or secondary outcome.

**S.R.No**.	**References**	**Population**	**Instruments**	**EFT (*n*) and sessions**	**Control/s (*n*) and sessions**	***p* (EFT)**
1	Church et al. (2018c)	16 veterans with PTSD	ISI	(*n* = 8) 1 sessions/week for 10 weeks	TAU (*n* = 8)	*p* = 0.005 after 10 sessions
2	Church et al. (2016)	21 veterans at risk of PTSD	ISI	(*n* = 12) 6 sessions + TAU	WL group: (*n* = 9) TAU	*p* = 0.004
3	Church et al. (2013)	59 veterans with PTSD	ISI	(*n* = 30) 6 sessions	SOC/WL (*n* = 29)	ISI noted *p* < 0.0001 at 6-month follow-up
4	Babamahmoodi et al. (2015)	28 chemically pulmonary injured veterans	GHQ and Immunological tests	(*n* = 14) Eight 90-min weekly group sessions + 2 times daily home practices	WL control group (*n* = 14)	Anxiety/insomnia *p < * 0.001
5	Lee and Kim (2015)	20 senior insomnia patients	GDS-K	(*n* = 10) Eight 1-h group EFT sessions twice a week for 4 weeks	Sleep Hygiene Education (SHE) (*n* = 10) 8 sessions	No significant difference between EFT and SHE in PSQI score (5-week *p* = 0.818 and 9-week *p* = 0.047). SS scores: 5th week (*p* = 0.040) 9th week (*p* = 0.010)
6	Souilm et al. (2022)	60 elderly insomnia patients	PSQI	(*n* = 30) Eight 1-h group EFT sessions twice a week for 4 weeks	Sleep Hygiene Education (*n* = 30) 8 sessions	*p* = 0.005

GDS-K, Geriatric Depression Scale in Korea; GHQ, General Health Questionnaire; ISI, Insomnia Severity Index; ISS, Insomnia Severity Scale; PSQI, Pittsburgh Sleep Quality Index; SS, Sleep Scale; SOC, standard of care; TAU, treatment as usual; WL, wait-list. Sr. No., systematic review number.

A pilot study of 10 geriatric patients noted a similar reduction in insomnia, along with decreases in anxiety and depression and an increase in life satisfaction (Lee et al., 2011). This led to an RCT conducted with 20 participants that compared EFT to an active control, Sleep Hygiene Education (SHE; Lee et al., 2013). It demonstrated significant reductions in depression and insomnia in both treatment groups, with EFT superior to SHE.

However, a second RCT comparing EFT to SHE found that while both were effective for insomnia, SHE was superior (Souilm et al., 2022). After the intervention, 73.3% of the EFT group had good sleep quality, compared to 100.0% in the SHE group (*p* = 0.005). The median score for depression was lower in the SHE group (*p* < 0.001) while there was no difference in life satisfaction.

Insomnia was also assessed in an RCT of a stress management program offered to lawyers from the Athens Bar Association (Christina et al., 2016). While it found significant reductions in both quality of sleep and insomnia symptoms, it is not included in Table 13 because it combined a variety of methods, so the results were not due solely to EFT.

Insomnia is related to stress and to the regulation of the autonomic nervous system. The improvements found in the RCTs in Table 13 demonstrate a robust association between a reduction in stress symptoms following EFT treatment and decreases in insomnia.

#### Weight loss, cravings, and binge eating

Numerous RCTs have examined the use of EFT for weight loss and food cravings, summarized in Table 14. Among adolescents, EFT is an effective treatment strategy for increasing healthy eating behaviors, self-esteem, and compassion, as well as improving associated weight-related psychopathology (Stapleton et al., 2016c). It has demonstrated effects comparable with CBT in the treatment of food cravings among obese adults (Stapleton et al., 2017). In a study of a 6-week online EFT program, significant improvements were found for body weight (*p* < 0.001), behaviors to restrain eating (*p* = 0.025), and the association of food with reward (*p* = 0.018). Participant weight decreased an average of 1 pound per week during the course and 2 pounds per month between pretest and 1-year follow-up (Church et al., 2018a).

**Table 14:** Weight and eating behavior RCTs.

**S.R.No**.	**References**	**Population**	**Instruments**	**EFT (*n*) and sessions**	**Control/s (*n*) and sessions**	***p* (EFT)/*d*/%**
1	Stapleton et al. (2017)	83 overweight or obese adults	PHQ	(*n* = 51) 1 session/week for 8 weeks	CBT (*n* = 34) 1 session/week for 8 weeks	Body weight (*p < * 0.001) Restraint (*p* = 0.025) Power of food in external environment (*p* = 0.018)
2	Stapleton et al. (2019b)	451 obese adults	FCI, SA-45, Anthropometric Measures, SRWWM, SUD	(*n* = 314) 1 session/week for 8 weeks	WL Group (*n* = 137)	Weight (post): *p* = 0.002 Food craving pre and post: *p < * 0.001 (stayed same at 12 months) Power of food pre and post diff: *p < * 0.001 Restraint capacity: *p < * 0.001 Pre and post BMI measurement diff: *p* = 0.002
3	Stapleton et al. (2020a)	357 bariatric surgery patients	BMI, TFEQ-R18, FCI, RSE,	(*n* = 107) PPBP kit in addition to an 8-week online self-paced EFT treatment	1. PPBP (*n* = 109) Asked to follow the PPBP kit for 8 weeks 2. TAU (*n* = 127)	Emotional eating (−16.33%) Uncontrolled eating (−9.36%) Self-esteem (+4.43%)
4	Stapleton et al. (2011)	96 overweight or obese adults	FCI, SA-45, Anthropometric Measures. SRWWM, SUD	(*n* = 49) 1 session/week (2 h) for 4 weeks	WL group (*n* = 47)	Food craving *p < * 0.001 Power of food *p < * 0.001 Restraint *p < * 0.001
5	Stapleton et al. (2012)	96 overweight or obese adults	FCI, SA-45, Anthropometric Measures. SRWWM, SUD	(*n* = 49) One 2-h session/week for 4 weeks	WL group (*n* = 47)	*p < * 0.05 for weight, BMI, food cravings, subjective power of food, craving restraint, and psychological coping for EFT participants from pretest to 12 months
6	Stapleton et al. (2016c)	44 college students	FCI, SA-45, Anthropometric Measures, SRWWM, SUD	(*n* = 14) 1 session/week for 6 weeks	WL group (*n* = 12)	There were clinically valid decreases in the psychological distress scores (but not a statistical significance). Results also indicated the students had significantly higher self-esteem and also self-compassion scores after the program.
7	Stapleton et al. (2016a)	83 overweight or obese adults	PHQ	(*n* = 51) 1 session/week for 8 weeks	(*n* = 34) CBT 1 session/week for 8 weeks	Anxiety: Across time *p < * 0.001, *p* = 0.002 from pre intervention to 6- and 12-month follow-up. No significant changes across time for CBT group. Depression: No significant differences for both groups at postintervention. At 6 months and 12 months, EFT (*p* = 0.16 and *p* = 0.116) and CBT (*p* = 0.246 and *p* = 0.124).
8.	Glisenti et al. (2021)	21 binge eating participants	DSM-5, SCID-5-RV, EDEQ, BES	(*n* = 10) 12 weekly 1-h sessions	EFT WL (*n* = 11)	Objective binge episode: *p* = 0.17, *d* = 0.98 Binge episode days: *p* = 0.001, *d* = 1.39 Binge eating psychopathology: *p* = 0.003; *d* = 0.62
9	Stapleton et al. (2019a)	15 overweight or obese adults	FCI	(*n* = 10) 1 session/week for 4 weeks	EFT WL (*n* = 5)	Food craving *p* = 0.031

BES, Binge Eating Scale; BMI, body mass index; CBT, Cognitive Behavior Therapy; DSM-5, Diagnostic and Statistical Manual of Mental Disorders, 5th edition; EDEQ, Eating Disorders Examination Questionnaire; FCI, Food Craving Inventory; PHQ, Patient Health Questionnaire; PPBP, Portion Perfection for Bariatric Patients; RSE, Rosenberg's Self-esteem Scale; SA-45, Symptom Assessment−45; SCID-5-RV, Structured Clinical Interview for DSM-5–Research Version; SRWWM, self-reported weight and weight measurements; SUD, Subjective Units of Distress; TFEQ-R18, Three-Factor Eating Questionnaire-Revised 18; WL, wait-list. Sr. No., systematic review number.

In a pilot randomized clinical trial of obese adults that investigated the effect of Clinical EFT on brain activation in response to food craving stimuli using fMRI, findings indicated that EFT may decrease limbic region brain activity and reduce food related symptoms in overweight/obese individuals (Stapleton et al., 2019a). Brain scans revealed deactivation in the superior temporal gyrus, among the functions of which is multisensory integration, as well as the lateral orbito-frontal cortex. This structure contains the secondary taste cortex and is also essential for the control and organization of behavior. The mean score for food cravings was reduced by 18% for the EFT group compared to 5% in the control group, with gains maintained over time (*p* = 0.031).

Analyses of the online EFT weight loss program described earlier (Stapleton et al., 2019b) indicated significantly reduced scores for food cravings (−28.2%, *p* < 0.001), the power of food over behavior (−26.7%, *p* < 0.001), depression (−12.3%, *p* < 0.001), anxiety (−23.3%, *p* = 0.005), and somatic symptoms (−1.6%, *p* < 0.001). Gains were maintained on 2-year follow-up. When EFT was added to a program called Portion Perfection for Bariatric Patients (PPBP), emotional eating decreased, with results maintained on 6-month follow-up (Stapleton et al., 2020a). A comparison of a brief 4-week (8-h) program vs. an 8-week (16-h program) yielded significant reductions in all measures for both intervention lengths (Stapleton and Chatwin, 2018).

Binge-eating disorder (BED) is the most prevalent of all the eating disorders (Kornstein et al., 2019). In a pilot RCT for BED (Glisenti et al., 2021), all participants experienced reliable recovery from binge-eating psychopathology and a significant decrease in binge-eating frequency. Emotion regulation and psychological conditions improved significantly.

Avery Lane for Women is an addiction clinic located in Novato, California. Data from 123 clients in their Rehabilitation Program were collected over a 3.5-year period (Popescu, 2021). Depression scores reduced from 79% at intake to 16% during the final contact point, which was usually a few months after completion of treatment (*p* < 0.001). Anxiety scores dropped from 73 to 8% (*p* < 0.001), trauma symptoms from 76 to 30% (*p* < 0.001), suicidality from 53 to 11% (*p* < 0.001), binge eating from 33 to 11% (*p* < 0.01), and compensatory eating disorder behaviors from 41 to 11% (*p* < 0.074). Instead of the usual pattern of relapse after leaving treatment, the Avery Lane study identified long-term maintenance in the majority of patients.

Among older studies, an RCT found that EFT improved dysfunctional restraint behaviors (Stapleton et al., 2011) and that, in the year following an EFT weight loss program, participants lost an average of 11.1 pounds (Stapleton et al., 2012). In the health care workers study summarized previously (Church and Brooks, 2010), cravings for substances such as chocolate, sweets, and alcohol were reduced by 83% in a single EFT session (*p* < 0.001). An uncontrolled study of clients in a 6-week online weight loss program found a 12-pound weight reduction during the 6 weeks of the program, followed by a further 3-pound drop in the ensuing 6 months (*p* < 0.001; Church et al., 2022).

Two RCTs compared EFT to CBT (Stapleton et al., 2016a, 2017). In the 2016 study, both CBT and EFT were found to be vital adjunct tools in a multidisciplinary approach to managing obesity. Both approaches demonstrated comparable efficacy in reducing food cravings, responsiveness to food in the environment (power of food), and dysfunctional dietary restraint. Both EFT and CBT normalized participant scores to the same level as a nonclinical community sample. In the 2017 study, both the EFT and CBT groups showed improvement on psychological metrics, with most gains maintained over time. The authors of these two studies concluded that EFT is comparable to Gold Standard approaches such as CBT.

An Egyptian study measured cravings in 90 patients diagnosed with substance use disorders at a psychiatric hospital in Alexandria (Balha et al., 2020). Significant improvements in somatization, obsessive-compulsive behaviors, interpersonal sensitivity, depression, anxiety, hostility, phobic anxiety, paranoid ideation, and psychoticism were identified after EFT, as well as a reduction in cravings (*p* < 0.001). This and the Avery Lane study are notable because, rather than enrolling research subjects, they studied populations of patients *in vivo* in clinical settings.

Introducing EFT into the treatment of food and substance use disorders allows the recovery process to be augmented by somatic procedures that strategically impact the neurological foundations of emotions, thought, and behavior in ways that facilitate desired changes. Brain imaging studies suggest that the demonstrated effectiveness of the approach is related to the way specific acupuncture points, when stimulated, send activating or deactivating signals to brain areas involved in targeted emotional and cognitive processes (Feinstein, 2016). Whether considered from a biological or behavioral perspective, ample evidence demonstrates that EFT is an extraordinarily effective non-drug treatment for food and substance use disorders. It warrants adoption as a first-line treatment of choice in clinical settings.

### Sports, academic, and professional performance, and positive emotions

Mental health studies typically measure reductions in conditions such as anxiety, depression, and PTSD. The focus of performance studies is different. They take individuals who are already performing at a certain level and seek to determine if an increase in performance is associated with an intervention. There is a wide range of performance metrics from points scored in sports, to test scores in students, to productivity in professional occupations. Performance can also be measured by decreases in symptoms such as anxiety and stress and correlated with increases in measures of success. One of the earliest uses of EFT was for sports performance, and in the past decade, athletes from school tennis players to Olympic runners to professional baseball players have been recorded using EFT. Table 15 summarizes RCTs of EFT for performance.

**Table 15:** Sports and other performance RCTs.

**S.R.No**.	**References**	**Population**	**EFT sessions**	**Control(s)**	***p* (EFT)/*d*/%**
1	Church (2009)	26 college basketball players	(*n* = 13) One 15-min session	Control group (*n* = 13) received an encouraging talk	A statistically significant difference between the treatment groups was found (*p < * 0.03). EFT Group: 21% better individually in free throws. Control group scored an average of 17% lower (*p < * 0.028).
2	Baker (2010), a reexamination of Church (2009) study	26 college basketball players	(*n* = 13) One 15-min session	Control group (*n* = 13) received an encouraging talk	This reanalysis of Church (2009) produced the same conclusion as above.
3	Llewellyn-Edwards and Llewellyn-Edwards (2012)	26 female soccer players	(*n* = 13) 10 min EFT session	(*n* = 13) Normal soccer technique coaching	Increase in free kick accuracy (*p < * 0.05)
4	Sezgin and Özcan (2009)	70 high school students with high anxiety	EFT 1 session + self-treatment at home (*n* = 35)	PMR 1 session + self-treatment at home (*n* = 35)	EFT and PMR were effective in decreasing test anxiety; reduction in TAI scores was greater in EFT group (*p < * 0.05)

PMR, Progressive Muscular Relaxation. Sr. No., systematic review number.

Two RCTs have examined EFT's efficacy for sports performance. One measured the difference in basketball free throw percentages between an EFT and a placebo control group and found a performance difference of 38% after a brief session (Church, 2009; Baker, 2010). Another found similar benefits for soccer free kicks (Llewellyn-Edwards and Llewellyn-Edwards, 2012). A case study of golf performance found stress-related errors decreasing after EFT (Rotherham et al., 2012). A 20-min EFT session was found to increase confidence and decrease anxiety in an uncontrolled study of female college-aged athletes (Church and Downs, 2012).

Several studies summarized in the previous paragraphs examined the application of EFT to professional performance issues such as public speaking anxiety and test anxiety and found improvements (Sezgin and Özcan, 2009; Jones et al., 2011). The NHS service evaluation performed by Boath et al. (2014a) examined patient self-esteem and mental wellbeing and found that both improved significantly (*p* < 0.001).

A non-randomized sample of 53 university students was given a public speaking assignment known to generate anxiety, followed by a brief EFT session. Significantly reduced anxiety was observed in those in the EFT group, accompanied by an increase in focus and calmness (Boath et al., 2013). Similar effects were observed in social work students, who characterized EFT as calming and relaxing (Boath et al., 2017). A similar reduction in anxiety was found in an RCT assessing a group of students preparing for university entrance exams (Sezgin and Özcan, 2009). Their test scores increased, though the improvement did not reach the threshold for statistical significance. The uncontrolled study of female college athletes found that confidence increased as anxiety decreased (Church and Downs, 2012).

Nursing students had reduced stress 4 weeks after learning EFT (*p* < 0.005; Patterson, 2016). They also exhibited decreases in both state and trait anxiety (*p* < 0.05), with students reporting lowered stress and somatic symptoms.

In academic settings, EFT is an efficacious intervention to reduce anxiety significantly in high-ability adolescents (Gaesser and Karan, 2017), and in recent years, teachers have underscored the importance of formal training in EFT for stress and anxiety management among students and staff in school settings (Gaesser, 2020). The Canadian study reviewed previously (Ledger, 2019) found that a large majority of students believed EFT should be taught in schools.

A number of studies both published and in press have used a Likert scale to measure happiness. They generally find that happiness levels increase as negative emotions subside in intensity (Bach et al., 2019). Positive emotions typically have an inverse relationship with negative ones. Performance, whether in athletic competitions, academic tests, interpersonal relationships, or business productivity, is generally observed to be inhibited by excessive stress. In the studies summarized previously that contain a measure of positive emotions, this effect is found to accompany EFT treatment.

### Physiological mechanisms of action

Outcome studies, which compare patient results before and after treatment, are clearly the most clinically important type of research. However, while showing *that* a treatment works allows it to be designated as an “evidence-based” practice, showing *how and why* it works allows us to understand the physiological changes that underlie its clinical benefits. Such studies provide objective physiological evidence to augment subjective self-report. RCTs that illuminate EFT's physiological mechanisms of action are presented in Table 16.

**Table 16:** RCTs illuminating EFT's mechanisms of physiological action.

**S.R.No**.	**References**	**Population**	**Instruments**	**EFT (*n*) and sessions**	**Control/s (*n*) and sessions**	***p* (EFT), %, *d***
1	Church et al. (2012c)	83 nonclinical subjects	SA-45,| Salivary cortisol assay	(*n* = 28) One 50-min session	Supportive interview based on CBT (*n* = 28) One 50-min session NT (*n* = 27)	EFT: Cortisol (−24.39%, *p* < 0.03) Anxiety (−58.34%, *p < * 0.05) Depression (−49.33%, *p < * 0.002) Overall severity of symptoms (−5.5%, *p < * 0.001) Symptom breadth (−41.93%, *p < * 0.001)
2	Stapleton et al. (2020b)	53 nonclinical subjects	SA-45, Salivary cortisol assay	(*n* = 17) One 60-min group intervention	Psychoeducation (*n* = 17) One 60-min group intervention NT (*n* = 17)	EFT: Cortisol (−43.24%, *p < * 0.05)
3	Church et al. (2018c)	16 veterans with PTSD	Blood gene expression assay SA-45, HADS, ISS, SF-12v2, Rivermead	(*n* = 8) One session/week for 10 weeks	TAU only (*n* = 8)	EFT: PTSD symptoms (−53%, *p < * 0.00001) Differential expression of six genes (*p < * 0.05)
4	Church and Nelms (2016)	47 participants with frozen shoulder	SA-45, MPRS, PST	(*n* = 16) One 30-min EFT session with acupoint tapping	DB (*n* = 18) One 30-min session WL (*n* = 13)	EFT: Psychological symptoms (*p < * 0.001) Cohen's *d* = 0.9 for anxiety and pain, and *d* = 1.1 for depression
6	Rogers and Sears (2015)	56 college students	Nine common stress symptoms	(*n* = 26) 15–20 min single group session	Sham Acupressure group (*n* = 30) 1 session	EFT: Stress symptoms reduced −39.3% (*p < * 0.001)
7	Yount et al. (2019)	16 veterans with PTSD	SA-45, mRNA test	(*n* = 8) 10 60-min sessions	NT (*n* = 8)	Decrease in expression levels of 2 depression-linked microRNAs: let-7b (*p* = 0.021) and let-7c (*p* = 0.015)

AEQ, Achievement Emotions Questionnaire; CBT, Cognitive Behavior Therapy; DB, Diaphragmatic Breathing; HADS, Hospital and Anxiety Depression Scale; ISS, Insomnia Severity Scale; MPRS, Matheson Pain Rating Scale; NT, no treatment; PST, Positive Symptom Total; Rivermead, Rivermead Post-Concussion Symptoms Questionnaire; SA-45, Symptom Assessment−45; SF-12v2, Short Form−12 item, version 2; TAU, treatment as usual. Sr. No., systematic review number.

Three studies have used electroencephalogram (EEG) to examine the brain wave frequencies of participants before and after EFT. Swingle et al. (2004) compared the EEG readings of auto accident victims before and after they learned EEG and found a reduction in the frequencies associated with PTSD. Lambrou et al. (2003) used acupressure tapping with claustrophobics, comparing them with a non-claustrophobic group, and found an increase in theta EEG frequencies associated with relaxation. Using electromyography (EMG), they also found significant relaxation of the trapezius muscle. Anxiety also declined and participant gains were maintained on 2-week follow-up. Swingle (2010) found EFT to be beneficial in the treatment of seizure disorders.

The effect of acupressure as used in EFT echoes that found in studies of acupuncture needling. In a 10-year research program conducted at Harvard Medical School, fMRI studies demonstrated that the needling or electronic stimulation of acupoints consistently produced changes in activation in the hippocampus, amygdala, and other brain areas associated with fear and pain (Hui et al., 2005; Napadow et al., 2007; Fang et al., 2009).

If EFT is regulating the body's stress response and the hypothalamus-pituitary-adrenal (HPA) axis, then it is also logical to look for changes in stress hormones such as norepinephrine (adrenaline) and cortisol. To test this hypothesis, a triple-blind RCT examined the cortisol levels of 83 normal subjects before and after an hour of EFT (Church et al., 2012c). A second treatment group received a supportive interview while a control group simply rested. Comparison of the three groups revealed significant reductions in cortisol in the EFT group compared to the other two groups (*p* < 0.03). A statistically significant relationship between the reduction in psychological conditions such as anxiety (−58%), and cortisol (−24.4%) was also identified.

In a direct replication of Church et al. (2012c), an RCT to reexamine the effect of EFT on stress biochemistry (Stapleton et al., 2020b) examined changes in stress biochemistry and psychological distress symptoms. It randomly allocated 53 participants to one of three 60-min group interventions: EFT, PE, and NT. The EFT group's reduction in cortisol (−43.24%) was significantly different from that of the PE (−19.67%) or NT group (2.02%) (*p* < 0.05). In the section on performance above, the specific results of the Bach et al. (2019) study are described. It found significant improvement across a wide spectrum of biomarkers, including immune factors, heart rate, cortisol, and blood pressure. The Church et al. (2018c) and Maharaj (2016) studies found significant changes in gene expression, while Yount et al. (2019) identified microRNAs associated with epigenetic changes after EFT treatment.

Several studies have used fMRI to measure changes in brain activity before and after tapping. In an independent German investigation, Wittfoth et al. (2020) exposed 17 participants to disgust- and fear-inducing images. Brain regions involved in emotion and information processing were upregulated when viewing the images. But when participants alternated their attention between the images and tapping, those same regions were downregulated. The investigators found that this “bifocal” activity was effective for “re-organizing the underlying neural pathways” (p. 1).

The same research team investigated tapping in participants with fear of flying. Mean fear scores dropped (*p* < 0.001) and the percentage of participants meeting clinical criteria dropped from 89.7 to 24%. Differential regulation in several brain regions, including the amygdala, hippocampus, and temporal pole, was found (Wittfoth et al., 2022). In a French study using magnetoencephalography (MEG) to study the brain activity of a subject with fear of flying, investigators found that EFT downregulated regions implicated in the fear response (Di Rienzo et al., 2018). It also increased activity in the prefrontal areas active in executive control and the management of emotions.

In an Australian study, 15 obese adult patients self-administered an EFT protocol at regular intervals over a 4-week period (Stapleton et al., 2019a). Foods that activated areas of the brain associated with hunger and craving prior to the program produced less activation in those areas at the end of the program, as shown by fMRI. This decreased activation corresponded with a diminished desire for those foods.

In the study of EFT for chronic pain patients (Stapleton, 2022), 24 adults were allocated to a 6-week online group EFT treatment and underwent resting-state fMRI pre and postintervention. A repeated measures MANOVA indicated significant differences in the levels of pain severity (−21%), pain interference (−26%), quality of life (+7%), somatic symptoms (−28%), depression (−13.5%), anxiety (37.1%), happiness (+17%), and satisfaction with life (+8.8%) from pre to posttest. fMRI analysis showed significantly decreased connectivity between the medial prefrontal cortex (a pain modulating area) and bilateral gray matter areas in the posterior cingulate cortex and thalamus, both areas being related to the modulating and catastrophizing of pain. There were no brain areas that showed significantly increased connectivity.

Improvements in mental health after therapy can be reflected in reduced levels of cortisol and regulation of the genes that code for such hormones (Feinstein and Church, 2010). Scientists studying epigenetics emphasize the role stress and emotion play in gene expression (Eley and Plomin, 1997; Fraga et al., 2005; Jirtle and Skinner, 2007; Church, 2010). The research into EFT's physiological mechanisms of action demonstrates this relationship. EFT has now been associated with changes in brain-wave activity (Lambrou et al., 2003; Swingle et al., 2004; Swingle, 2010), stress hormone levels (Church et al., 2013; Stapleton et al., 2020b), gene expression (Maharaj, 2016; Church et al., 2018c), brain region activation (Wittfoth et al., 2020, 2022), epigenetic microRNA activity (Yount et al., 2019), and biomarkers such as heart rate, immunity, and blood pressure (Bach et al., 2019). A wide range of physiological mechanisms, including epigenetic, endocrinal, cardiovascular, immunological, and neurological components, have thus been found to be associated with EFT treatment.

### Is acupoint tapping an active ingredient in EFT?

EFT's “Setup Statement” is an essential part of the “Basic Recipe.” The Setup Statement has two parts. One is a statement of the client's presenting problem, prefaced with, “Even though I have this problem…” while tapping on a specified point on the small intestine meridian. They repeat the name of the problem while tapping on the other points. This focus on the problem is reminiscent of the techniques practiced in Prolonged Exposure (PE) and other exposure therapies.

The second half of the Setup Statement directs the client toward acceptance of conditions as they are: “… I deeply and completely accept myself.” This cognitive reframe is akin to certain techniques used in cognitive therapies that seek to modify dysfunctional client cognitions and emotional responses to events. In a review of therapies for PTSD, the US government's Institute of Medicine [IOM] (2008) found that therapies using exposure and cognitive shift were efficacious. EFT's Setup Statement draws from elements of these two established therapies.

The third ingredient used by EFT is tapping on points used in acupuncture and acupressure (acupoints). Is this component of EFT an active ingredient or is EFT's efficacy dependent solely on the exposure and cognitive components it shares with other therapies?

During the first meta-analysis testing the efficacy of acupressure tapping in the treatment of psychological distress (Gilomen and Lee, 2015), it was concluded that due to methodological shortcomings in the studies examined, it was not possible to determine whether EFT's effects were due to acupoint stimulation or simply due to treatment elements common to other therapies.

A meta-analysis was performed to address this question (Church et al., 2018b). It examined six studies in which an active control, such as diaphragmatic breathing or sham acupoints, was used in place of tapping on actual acupoints. Studies (*n* = 403) were assessed using the APA standards for quality control, and three (*n* = 102) were identified as meeting them. Pretest vs. posttest EFT treatment showed a large effect size, Cohen's *d* = 1.28 (95% confidence interval [CI], 0.56–2.00) and Hedges' *g* = 1.25 (95% CI, 0.54–1.96). Acupressure groups demonstrated moderately stronger outcomes than controls, with weighted posttreatment effect sizes of *d* = −0.47 (95% CI, −0.94–0.0) and *g* = −0.45 (95% CI, −0.9–0.0). This meta-analysis indicated that the acupressure component was an active ingredient and outcomes were not due solely to placebo, nonspecific effects of any therapy, or non-acupressure components.

This meta-analysis was subsequently challenged (Spielmans et al., 2020), which led to the discovery of statistical errors in the original calculation, primarily incorrect standard deviations, and the correction of these (Church et al., 2020b). The revised analysis found a slightly more robust effect for EFT's long-term effects than the original analysis. The cumulative fixed effects Hedges' *g* was found to be 0.73 (95% CI = 0.42–1.04, *p* < 0.0001). The corresponding random effects Hedges' *g* is 0.74 (95% CI = 0.34–1.13, *p* < 0.0001). This result echoed the studies themselves, every one of which indicated that tapping made a contribution to EFT's reported effects.

Collectively, these results demonstrate that EFT's acupoint stimulation is an active ingredient. It is supported by the Harvard studies cited earlier that used fMRI to measure the effects of acupuncture on the areas of the brain associated with fear (Hui et al., 2005; Napadow et al., 2007; Fang et al., 2009). These studies uniformly report acupuncture to produce rapid regulation of these brain regions, as do EFT fMRI studies (Stapleton et al., 2017; Wittfoth et al., 2020, 2022). When the established protocols drawn from exposure and cognitive therapies are paired with acupressure, their effects are enhanced.

### Borrowing benefits: EFT as group therapy

During the early development of EFT, therapists reported lower levels of stress and burnout than they had experienced administering conventional treatments. This led to the hypothesis that tapping on oneself while demonstrating tapping to others or witnessing tapping on others while tapping on oneself diminished distress. This phenomenon is known as “Borrowing Benefits” (Craig, 2008/2010). A series of studies has measured the efficacy of Borrowing Benefits for psychological and physical symptoms, especially when performed in group settings.

The first such study was performed by Rowe (2005) who examined the psychological symptom levels of 259 participants in a weekend EFT workshop, 102 of whom provided complete data. He found reductions in nine common conditions such as anxiety and depression, with participant gains maintained on follow-up.

Group application of EFT was also found to reduce psychological symptoms such as anxiety in a group self-identified with addiction issues (Church and Brooks, 2013). The health care workers study cited previously (Church and Brooks, 2010) found similar results. At follow-up, the symptom levels of participants who had used EFT frequently were lower than those who had not. It found greater improvements in more frequent users. One of that study's replications (Palmer-Hoffman and Brooks, 2011) identified similar effects from Borrowing Benefits (*p* < 0.001).

PTSD symptoms were examined in a study of 218 veterans and spouses who used Borrowing Benefits during 7-day group retreats (Church and Brooks, 2014). On pretest, 82% of veterans and 29% of spouses met the criteria for clinical levels of PTSD symptoms. After the retreat, at 6-week follow-up, only 28% of veterans and 4% of spouses were PTSD-positive (*p* < 0.001). The study compared the results of five such retreats, reporting in effect the results of five individual substudies. Similar symptom declines were noted in all five groups.

At a 2-day EFT workshop (Church and House, 2018), Borrowing Benefits was associated with reductions in anxiety, depression, and PTSD (*p* < 0.03), with participants maintaining their gains at 6-month follow-up (*p* < 0.02).

Though these were uncontrolled studies, several RCTs also utilized Borrowing Benefits in a group therapy design. The study of college students with depression (Church et al., 2012a) offered the EFT intervention in four group counseling sessions. A study of depression in weight loss subjects also taught participants EFT in group classes (Stapleton et al., 2013). In two of the studies of sports performance (Church, 2009; Llewellyn-Edwards and Llewellyn-Edwards, 2012), the EFT cohort received at least part of the intervention as a group. EFT was also provided in groups of 10 in the Congo RCT of traumatized females and found to be as effective as CBT in reducing PTSD, anxiety, and depression (Nemiro and Papworth, 2015). The insomnia RCT also administered both EFT and active control in group format (Lee et al., 2013).

Other populations in which Borrowing Benefits has demonstrated efficacy include business executives (Church and David, 2019), chronic pain patients (Stapleton et al., 2016b), psoriasis patients (Hodge and Jurgens, 2011), students with public speaking anxiety (Madoni et al., 2018), people with food cravings (Stapleton and Stewart, 2020), nurses (Dincer and Inangil, 2020), and Hwabyung patients (Song et al., 2014; Kwak et al., 2020), to name a few.

These studies are notable in that significant reductions in symptoms occurred when EFT was delivered as group therapy, as opposed to individual counseling. The number of recent US veterans with PTSD from wars in the Middle East has been estimated at 500,000 (Thompson, 2015). Each veteran with PTSD is estimated to cost society $1,400,000 (Kanter, 2007), implying a social cost of about a trillion dollars to treat veterans of these and earlier conflicts. Group approaches such as Borrowing Benefits, which produce symptom reductions without the need for lengthy courses of individual psychotherapy or chronic use of prescription drugs, are efficient and cost-effective.

### Disaster relief

Feinstein (2022) provided a summary of research examining the use of energy psychology techniques such as tapping after catastrophic events, including mass shootings, genocide, ethnic warfare, earthquakes, hurricanes, tornadoes, floods, wildfires, and the COVID pandemic. The studies show strong outcomes for energy psychology as psychological first aid in the days or weeks after a disaster and in later treatment of trauma-based psychological problems. Rapid relief and long-term benefits are corroborated across studies.

### Virtual EFT

Not only are in-person groups effective, virtual administration of EFT can also yield favorable outcomes. A study comparing a virtual EFT group workshop with an in-person one found significant depression symptom reduction in both (Church and Clond, 2019). Several of the weight loss studies referenced previously were conducted entirely online, and those that used group interaction all demonstrated clinically and statistically significant results.

The tapping app study reviewed in the introduction to this paper is noteworthy. Significant decreases in anxiety and stress were measured after brief self-tapping sessions (Church et al., 2020a). A meta-analysis found similar outcomes for several psychological conditions whether the interventions were conducted virtually or in person (Fernandez et al., 2021).

The model prevalent in the 20th century of a client sitting in a practitioner's office and receiving in-person treatment is being displaced by virtual interactions in the 21st century. Future studies will evaluate the efficacy of virtual tapping, and platforms and programs to deliver Clinical EFT virtually are likely to proliferate. These liberate treatment from the constraints of time and place, since they can be utilized by clients at their convenience. This will make the therapeutic benefits of tapping available to wider demographic segments and speed its proliferation.

### Simultaneous symptom reduction

Most psychological research seeks to isolate a single condition and excludes multiple diagnoses (Seligman, 1995). However useful this exclusion criterion might be for research, most patients present with a complex of disorders rather than a single one (Gorman, 1998). The studies of EFT described in this paper are notable in this regard. Not only do multiple psychological conditions decline simultaneously, but multiple physiological diagnoses do as well. All organ systems are affected by stress, and when EFT reduces stress, the results are pervasive in body, mind, and emotions. Future research and treatment is likely to focus on the multidimensional benefits of Clinical EFT, rather than attempting to evaluate its utility in a single diagnostic category.

### Safety

Cumulatively, over 2,000 subjects have participated in trials of EFT without a single adverse event being reported, indicating a high degree of safety. EFT also appears to be safe when administered by a therapist or life coach or self-administered. Therapists treating victims of childhood sexual abuse preferred energy psychology treatments such as EFT over talk therapy because they found the risk of abreaction low with the former (Schulz, 2009). Mollon (2007) reports a general reduction of client distress during acupoint tapping, while Flint et al. (2005) remark on the absence of abreactions during energy psychology treatments. Most studies of EFT have been performed after Institutional Review Board (IRB) review. IRB procedures require that studies be designed and conducted in a manner that protects human subjects, including a requirement that participants be monitored for adverse events. As mentioned previously, the US Veterans Administration has designated Clinical EFT a “generally safe therapy” (Church, 2017). These factors combine to indicate that the practice of EFT is not generally associated with the likelihood of harm.

### Replication studies

Replication studies have been performed for PTSD, depression, anxiety, phobias, cortisol, pain, and sports performance, as noted previously. See Table 17 for summaries. Two replications (Baker and Siegel, 2010; Salas et al., 2011) corroborated the findings of the Wells et al. (2003) phobia study. Two replications (Geronilla et al., 2016; Church et al., 2018c) found results similar to the first PTSD RCT (Church et al., 2013).

**Table 17:** Original and replication RCTs.

**Condition**	**Original**	**Replication**	**Results**
Phobia	Wells et al. (2003)	Baker and Siegel (2010)	EFT lowers phobia of small animals
		Salas et al. (2011)	
PTSD	Church (2014)	Geronilla et al. (2016)	EFT lowers symptoms of PTSD
		Church et al. (2018c)	
Cortisol	Church et al. (2012c)	Stapleton et al. (2020b)	EFT lowers cortisol
Depression	Chatwin et al. (2016)	Jasubhai and Mukundan (2018)	EFT lowers depression in MDD
Anxiety	Madoni et al. (2018)	Dincer et al. (2020)	EFT lowers anxiety related to public speaking
Sports Performance	Church (2009)	Llewellyn-Edwards (2013)	EFT improves performance

MDD, Major Depressive Disorder.

A replication (Stapleton et al., 2020b) of the cortisol study (Church et al., 2013) also found significant effects. Anxiety replications, most notably for public speaking anxiety, have shown that a single EFT treatment session is usually sufficient. Depression replications have also corroborated the findings of earlier trials (Chatwin et al., 2016; Jasubhai and Mukundan, 2018). A study of sports performance (Llewellyn-Edwards, 2013) replicated the results of an earlier study (Church et al., 2009).

A study examined psychological symptoms, pain, and cravings in 216 health care workers such as doctors, nurses, chiropractors, psychotherapists, and alternative medicine practitioners who attended a 1-day Clinical EFT workshop at one of five professional conferences (Church and Brooks, 2010). Though this was an uncontrolled study, it examined the five different groups separately, making it, in effect, five small studies. In addition, EFT was delivered by two trained and certified Clinical EFT practitioners. Despite their disparities, all five groups showed similar results.

A study was designed to both replicate these results and extend them by measuring the effects of treatment by a variety of trained and certified practitioners (Palmer-Hoffman and Brooks, 2011). With four different practitioners offering Clinical EFT sessions in various settings, similar outcomes were observed. The authors concluded that it was the Clinical EFT method itself, rather than the unusual gifts of any one practitioner, that was responsible for the observed effects. A similar design was used in the uncontrolled addictions study described previously (Church and Brooks, 2013), which again found the effects of Clinical EFT to be independent of practitioner.

As noted in the earlier discussion of the “replication crisis” in science, large and well-funded efforts to replicate key studies have been successful for only a minority of studies. The finding that the results of EFT studies have been replicated for anxiety, depression, PTSD, phobias, cortisol, sports performance, and practitioner efficacy is notable.

## Discussion

### Characteristics of clinical EFT

The body of literature summarized in this paper allows us to draw several conclusions about Clinical EFT. These include:

#### Regulation of stress

The epigenetic, hormonal, and neurological evidence demonstrates that Clinical EFT regulates the body's hypothalamic-pituitary-adrenal **(**HPA) axis and attenuates the stress response. Since stress recruits all the body's major organ systems, the reduction of stress produces pervasive physiological regulation.

#### Reliability

When applied with fidelity to the manual, Clinical EFT can be relied upon to produce the magnitude of change identified in the evidence base.

#### Durability

Long-term follow-ups demonstrate that when issues are resolved after tapping, gains are maintained over time.

#### Safety

Clinical EFT, both when self-administered and when administered by trained and certified practitioners, produces a reduction in affect and has not been associated with adverse events.

#### Speed

Clinical EFT works quickly. Treatment time frames range from one session for phobias to 10 sessions for PTSD. For some conditions, it is effective in time frames of 15 min or less.

#### Simultaneous reduction of multiple psychological conditions

Clinical EFT's stress-reduction capacity makes it effective at the same time in alleviating multiple psychological conditions, including phobias, PTSD, anxiety, and depression.

#### Physical symptom reduction

The amelioration of psychological stress has a wide range of physiological benefits. Clinical EFT has been shown to remediate physical symptoms associated with pain, insomnia, immunity, addictions, hypertension, autoimmune conditions, traumatic brain injury, and a variety of other diagnoses. However, the lack of RCTs and replications means that Clinical EFT cannot yet be considered an evidence-based treatment for these conditions.

#### Replicability

Clinical trials in which trained and certified practitioners apply the method with fidelity to *The EFT Manual* produce consistent results.

#### Generalizability

Clinical EFT has demonstrated efficacy with widely disparate demographic samples, occupational groups, diagnoses, and conditions. It has produced consistent results among independent teams of investigators and in multiple geographic locations, making its measured effects generalizable.

#### Trainability

Rather than requiring extraordinary personal healing abilities, the 48 techniques of Clinical EFT can be learned in a structured and supervised training program. Practitioners trained in the form of the method described in the manual are able to produce consistent results.

#### Self-help

Clinical EFT is safe and effective when self-applied as well as when it is practiced by trained professionals.

#### Group therapy

Clinical EFT's group therapy method, described in the manual as “Borrowing Benefits,” is effective in groups of various sizes and in virtual as well as in-person groups.

#### Virtual delivery

When EFT is delivered using virtual delivery platforms such as apps and online courses, initial evidence suggests efficacy comparable to in-person application. Future treatment and research options will extend the options for treatment delivery using artificial intelligence (AI), virtual reality (VR), and immersive reality (IR).

#### Public health impact

Clinical EFT's efficacy and ease of use makes it relevant to public health issues such as obesity, PTSD, disaster relief, stress, and addiction treatment. Clinical EFT is increasingly being adopted in primary care settings where it has the potential to produce pervasive improvements in public health.

#### Cost effectiveness

The brevity of treatment time frames required to produce symptom reduction, as well as Clinical EFT's efficacy when delivered using groups and virtual platforms, makes it a cost-effective treatment option.

### Limitations

This systematic review has several limitations. While the meta-analyses used standards such as the APA criteria as a quality control for the RCTs reported, this review did not screen RCTs through a similar quality control filter. Because the purpose of the review was to make the evidence base available and comprehensible to clinicians, rather than meet any set of technical standards, or perform a statistical analysis, all RCTs regardless of quality were reported.

A further limitation is that because of the large number of RCTs reported since the meta-analyses, and the even larger number of primary and secondary outcomes, all outcomes were not reported. Only those determined to be most clinically relevant were included.

Another limitation is that a further quality assessment such as a risk-of-bias (ROB) analysis of the included studies was not undertaken. The reason for this was twofold. One is that ROB was not included in most of the original meta-analyses. The second is that when ROB standards are applied to trials of psychotherapeutic methods, including EFT, the results are often misleading.

For instance, one ROB standard is: “2.2. Were carers and people delivering the interventions aware of participants' assigned intervention during the trial?” The answer in psychotherapy trials is virtually always “Yes” leading to a conclusion that there are “concerns of ROB.”

However, it is clearly impossible for a therapist trained in the psychotherapeutic intervention being evaluated to deliver that intervention to a study participant while remaining blind to the intervention being delivered. While this and other ROB standards are perfectly appropriate for drug trials, many do not transfer well to mental health studies and lead to an inappropriately large calculation of ROB when ROB is actually low.

## Future research and treatment directions

In the earlier iteration of this paper (Church, 2013a), future research directions were recommended. Over the past decade, progress has been made toward several of these, such as trials with larger Ns, institutional support for research, studies of virtual delivery platforms, evaluation of biomarkers, and measurement of Clinical EFT's stress-reduction effects on medical diagnoses. The large number of RCTs published since that time has greatly increased the evidence base, and as new studies are published, they appear on Research.EFTuniverse.com. Besides the studies published in English-language journals, a great deal of EFT research has occurred in non-Western countries. A recent review mentioned earlier identified 91 papers published in non-English-language journals (Freedom et al., 2022). Based on this literature, what are useful next steps for EFT research?

### Institutional trials in western countries

While many studies have been performed in association with universities, hospitals, and clinics, the majority of those published in English-language journals have been conducted in outpatient settings by private foundations. In contrast, most of those published in non-English-language journals have been conducted within institutions. Many of these studies have been undertaken in primary care settings (Freedom et al., 2022), suggesting that EFT is available in many countries. This is not the case in North America and Northern Europe. In the US, for example, not a single study has been undertaken by the Veterans Administration despite its mission of caring for veterans with PTSD. Studies within institutions such as large hospitals that are published in English-language journals will contribute toward a framework for institutional implementation of EFT.

### Virtual practitioner sessions

Virtual therapy is playing an ever-expanding role in delivering therapies of all types to clients. The COVID epidemic hastened the adoption of virtual therapy worldwide. Many therapists and coaches transitioned their practices from in-person to virtual sessions, and did not return to in-person practice afterward. Informal reports from EFT practitioners demonstrate the same pattern. A meta-analysis comparing the efficacy of virtual video with in-person therapy found no significant differences in outcomes for anxiety, depression, or PTSD (Fernandez et al., 2021).

Both clients and providers have become increasingly comfortable with, and proficient at, virtual platforms like Zoom and Facetime. Shortly before the pandemic, a platform called Stress Solution became available (MyStressSolution.com). It facilitates Clinical EFT video sessions between certified practitioners and clients. Clients log on, text an available practitioner, and initiate a session. The convenience of such virtual practitioner sessions makes Clinical EFT readily available. Because they can be offered across time zones and geographic locations, virtual video practitioner sessions also greatly expand access to efficacious treatments such as Clinical EFT.

### Integrative wellness studies

Despite half a century of evidence of their value to patients, behavioral interventions such as meditation, yoga, acupuncture, and EFT are rarely integrated into primary care. Future research should determine how to introduce patients to EFT effectively and encourage compliance with a health-promoting stress-reduction regimen. Apps such as the Tapping Solution and Stress Solution can be offered as outpatient stress-reduction options and their results evaluated. Pre and post surgery, patients could be offered in-person or virtual EFT to cope with stress and support healing. Between psychotherapy sessions, EFT can be applied as self-help, with its results quantified by research.

### On-demand sessions

Not only do virtual platforms like Stress Solution make EFT treatment available independent of location and time zones, they also make it possible to get interventions to clients when they require them most. For example, having a late-night session available to a client experiencing insomnia is more immediately useful than having to book a future appointment that will take place at a time when insomnia is no longer an immediate concern. Immediate access for a client who is suicidal has obvious benefits over an appointment-based system. For this reason, on-demand models and platforms are likely to proliferate, and by gathering data on these delivery systems, their efficacy can be measured.

### Accessibility

Virtual therapy platforms such as Stress Solution make Clinical EFT available to underserved populations. Examples of the latter include clients who are geographically remote from mental health services, clients who perceive a stigma to treatment, and clients who have physical handicaps that prevent them from traveling to treatment locations. Research can catalog the benefits of the availability of mental health services to such samples.

### Personalized medicine

The medicine of yesterday sought treatments that were generalizable to large populations. In the US, for instance, from the 1950s onward, oncology moved toward the enshrinement of chemotherapy, surgery, and radiation as standard care. In psychology, CBT became the one-size-fits-all recommendation in practice guidelines (e.g., Courtois et al., 2017). Today, advanced diagnostic tools allow treatments to be personalized to individuals. Genetic tests, for instance, identify alleles that allow individualized chemotherapy cocktails to be formulated for a single cancer patient.

Psychology is starting to recognize that combination therapies targeted to a particular client's neurological, developmental, and behavioral profile can be more effective than standardized approaches. As costs drop and assays become as noninvasive as a saliva sample, future studies will tailor psychotherapeutic approaches to a client's unique experience and track their success experimentally through frequent psychological and genetic assessments.

### Virtual and immersive reality

Treatments using virtual reality (VR) goggles have already established themselves as effective treatments for PTSD (Rizzo and Shilling, 2017). VR has also been used to treat a variety of other psychological conditions (Mishkind et al., 2017). The resolution of VR is increasing steadily, while immersive reality (IR) experiences simulate other senses such as touch and smell. Eventually, the technology is expected to merge into a holographic environment akin to the “holodeck” experience depicted in science fiction media. Futurists estimate that by the middle of this century the technology will have advanced to the point that it will be impossible for human beings to tell whether they are in a virtual or actual environment (Diamandis and Kotler, 2020). EFT can be used in conjunction with VR, even at the latter's current level of development. Exposure is one of the three cornerstones of Clinical EFT (the other two being cognitive framing and acupressure). The vivid reexperiencing made possible by VR makes it an effective prelude to tapping; future studies could evaluate this combination.

### Artificial intelligence

Almost all personalized coaching and counseling relies on an encounter between practitioner and client. Most of the efficacy of psychotherapy has been found to be due to the therapist, not the particular therapeutic method employed (Wampold and Brown, 2005). The training, skill, temperament, and insight of the therapist is paramount, as is the degree of emotional rapport with the client. Though Clinical EFT relies on 48 clearly defined techniques, practitioner skill plays a role, even during virtual EFT sessions like those at MyStressSolution.com. A live practitioner interacts dynamically with a client to understand issues and select from among the possible therapeutic approaches.

Yet advances in artificial intelligence (AI) mean that many client cues can now be interpreted and responded to by automated agents. AI programs translate the client's speech to text, rapidly analyze it, and craft a personalized response. Ellie, an onscreen AI therapist funded by the US Defense Advanced Research Projects Agency (DARPA), has found a surprisingly high level of acceptance by veterans as a therapeutic tool for PTSD (Gamble, 2020). A similar trajectory is conceivable for AI-based Clinical EFT sessions, with tapping scripts customized to the nuances of a client's words, just as human therapists today select from among large numbers of possible responses and techniques.

AI continues to improve and it is possible to contemplate an app that reads and interprets client facial expressions, tone of voice, and other cues to personalize therapeutic recommendations. Research can measure the efficacy of AI against live therapy and advances in technology can reasonably be expected to produce progressively better client outcomes.

### Online and virtual courses

Courses package EFT instruction into forms convenient to clients. A client might enroll for a specialized tapping course for weight loss, relationship skills, or financial literacy and be able to watch Borrowing Benefits videos, read text, and interact with exercises at any convenient time. Studies of two online courses, Naturally Thin You (NaturallyThinYou.com; Church et al., 2018a) and Skinny Genes (SkinnyGenesFit.com; Church et al., 2022), have demonstrated an association between tapping and weight loss, while the online fibromyalgia course (FibroClear.com; Brattberg, 2008) found a reduction in symptoms in two thirds of participants.

To date, two studies have made direct comparisons between an online course and in-person tapping. A study comparing an in-person Tapping Deep Intimacy workshop with an online course found similar outcomes for anxiety and relationship satisfaction but different demographic characteristics for subjects selecting the two delivery formats (TappingDeepIntimacy.com; Church and Clond, 2019). In the chronic pain study described previously (Stapleton, 2022), a subset of the sample (as the study is still ongoing) suggests that an online self-paced version of EFT achieved slightly better outcome in somatic symptoms, depression, anxiety, and panic disorder (*p* < 0.001) and that EFT delivered virtually may be as effective as consulting in person. Such comparisons of online courses to in-person experience represent a fruitful new area of research. Clinical EFT training is also available as a standardized online course based on the manual (TheTappingCourse.com) and this could be compared with in-person classes teaching the 48 techniques. If online tapping can produce effects comparable to in-person experiences, Clinical EFT will become convenient to extended demographic samples.

### Wireless mobile devices and apps

Emerging technologies like smartphones allow EFT to be used portably during times of heightened stress. In the first study of an app described in this paper (Church et al., 2020a), reductions of over 40% in anxiety and stress were measured in users of the Tapping Solution app. This large treatment effect made available at a time and place convenient to a client, with the privacy and convenience of a wireless mobile device (WMD) such as a tablet or smartphone, without any personalized or formal therapy, extends the reach of Clinical EFT to new treatment dimensions. If a client wants to engage the services of a certified practitioner, they can do so through the Stress Solution app (TappingApps.com). This is an app version of the Stress Solution website described above. Whether through automated software like the Tapping Solution app or personal practitioners sessions like the Stress Solution app, the availability of Clinical EFT on WMD and the data culled from such interactions are likely to extend the benefits of tapping to a wider demographic.

### Workplace and productivity assessments

The performance studies described in this paper link the stress-reducing effects of Clinical EFT to enhanced performance. Studies are now in progress measuring workplace productivity and the contribution Clinical EFT can make to both worker satisfaction and enhanced performance. If this effect is substantial, as hypothesized, tapping might become a routine part of organizational cultures.

### Primary care and medical treatment

The studies listed under the Physiological Conditions heading in this paper provide an early indication of Clinical EFT's potential role in healing, disease management, and medical practice. It is a non-pharmacological, natural, self-applied, and safe intervention, with stress-reduction effects that promote healing of many medical diagnoses and conditions. Most extant studies measure medical conditions as a secondary rather than primary outcome, but the coming decades are likely to see EFT explored as a primary treatment for these.

Modern medicine is particularly challenged by certain classes of disease. It has few remedies for fibromyalgia, psoriasis, multiple sclerosis, lupus, and other autoimmune conditions, and can at best provide palliative care. It has limited treatment options for cravings, addictions, and substance abuse, and relapse is the norm rather than the exception. It has been challenged by the increase in “lifestyle diseases” such as obesity, atherosclerosis, stroke, and diabetes.

Cancer, heart disease, hypertension, Alzheimer's, and many other diagnoses have been linked to stress, as has telomere shortening, cognitive decline, and loss of brain volume. Yet stress-reduction techniques such as meditation and tapping are rarely prescribed. Future trials could evaluate changes in all these conditions as primary outcomes. This would indicate for which conditions Clinical EFT might either be a first-line primary care treatment or an auxiliary behavioral option.

### Group scale studies

EFT is notable in its ability to improve symptoms when delivered to groups. However, the optimal group size for various conditions has not yet been empirically tested. Groups have ranged from over 250 participants (Rowe, 2005) to 10 (Church and Brooks, 2010). What is the minimum size to produce a group effect? What is the optimum size for each condition? Is there a group size at which the effects diminish? Research that answers these questions of scale will assist institutions using group therapy to optimize their use of Clinical EFT.

### Reduction of multiple symptoms

While research has tended to isolate conditions such as chronic pain or depression, EFT's ability to reduce both psychological and physiological symptoms in tandem strengthens the case for measuring symptom clusters. An approach to research that encourages the reduction of multiple symptoms simultaneously has clear clinical benefits.

### Client-centered focus

The APA Standards (Chambless and Hollon, 1998) advocate the use of valid and reliable instruments. These are usually self-reports that measure clinical change relevant to the client's direct experience. In contrast, the Tolin et al. (2015) standards emphasize observer-rated clinical diagnoses. Clinical EFT has been taught as a client-centered approach since its inception, and while observer-rated measures can certainly amplify understanding of a method's healing effects, future research should continue its client-centered focus.

### Biomarkers

The past decade has witnessed increasing use of biomarkers to measure the results of treatment with Clinical EFT. Future studies can expand the use of biomarkers such as oncogenes, telomerase, microRNAs, neurotransmitters, immunoglobulins, BMI, creatinine, T cells, heart rate, blood glucose, interleukins, hormones, blood pressure, HRV, cytokines, and C-reactive protein as objective measures of change.

## Conclusion

Clinical EFT, as validated in over 100 RCTs and outcome studies, has established itself as an efficacious treatment for both psychological and physical conditions. Its large and growing body of research shows it to be an evidence-based practice that is safe, fast, reliable, and cost-effective. Treatment time frames are brief, symptom improvements are durable and, both virtual and in-person sessions are effective. The objective effects of EFT treatment have been measured in physiological dimensions such as gene expression, brain wave synchrony, hormonal synthesis, cardiac function, immunity levels, and other biomarkers. Clinical EFT enjoys increasing professional acceptance as a first-line treatment in primary care. In the coming decades, it is likely to experience even greater public acceptance and be formally classified as a primary treatment for a variety of conditions.

## Author contributions

All authors listed have made a substantial, direct, and intellectual contribution to the work and approved it for publication.

## Conflict of interest

DC and PS derive income from publications and presentations on the therapeutic method described. The remaining authors declare that the research was conducted in the absence of any commercial or financial relationships that could be construed as a potential conflict of interest. The reviewer RH declared a shared affiliation with the authors DC and AV to the handling editor at the time of review.

## Publisher's note

All claims expressed in this article are solely those of the authors and do not necessarily represent those of their affiliated organizations, or those of the publisher, the editors and the reviewers. Any product that may be evaluated in this article, or claim that may be made by its manufacturer, is not guaranteed or endorsed by the publisher.
